# Supplementary material for: Integrated analysis reveals FOXA1 and Ku70/Ku80 as targets of ivermectin in prostate cancer
Source: Cell Death Dis. 2022 Sep 1;13(9):754. doi: 10.1038/s41419-022-05182-0 (PMC9436997; doi:10.1038/s41419-022-05182-0)
Supplement: Supplementary file 1 — Supplementary materials [file 41419_2022_5182_MOESM1_ESM.docx]

Supplementary Materials for

**Integrated analysis reveals FOXA1 and Ku70/Ku80 as targets of ivermectin in prostate cancer**

**Supplementary Figure Legends**

**Supplementary Fig. S1. (A)** Flow cytometry profiling of cell cycle distribution in LNCaP, C4-2 and 22RV1 cells treated with indicated concentrations of ivermectin following PI staining. **(B)** Flow cytometry profiling of cell cycle distribution in LNCaP, C4-2 and 22RV1 cells treated with indicated concentrations of ivermectin following PI and Annexin V staining.

**Supplementary Fig. S2. (A)** Representative images of colony formation assay of C4-2 and 22RV1 cells after treating with ivermectin for 48 hours. **(B)** Representative images of SA- β -Galactosidase staining (blue-green) of LNCaP, C4-2 and 22RV1 cells after treating with ivermectin for 48 hours.

**Supplementary Fig. S3. (A)** Ivermectin did not change the cell cycle distribution in DU145 cells for 48 hours. **(B)** Western blot analysis of PARP and γH2A.X in DU145 cells treated with ivermectin for 48 hours. **(C)** Ivermectin increased DNA damage. DNA fragments were shown as comet images in alkaline gel electrophoresis (Dox: Doxorubicin was used as positive control). The tail moment was used to quantify the DNA damage in the treatment of ivermectin for 48 hours. **(D)** Ivermectin did not change the cell cycle distribution in PC-3 cells for 48 hours. **(E)** Western blot analysis of PARP and γH2A.X in PC-3 cells treated with ivermectin for 48 hours. **(F)** The quantification results of Fig. 2G. **(G)** Western blot analysis of PARP and γH2A.X in 22RV1 xenograft tumor tissues treated with ivermectin.

**Supplementary Fig. S4.** The expression of AR or ARVs signaling target genes identified by RNA-seq in C4-2 **(A)** and 22RV1 **(B and C)** cells.

**Supplementary Fig. S5. (A)** GSEA showed that FOXA1 or FOXA1/AR consensus binding genes was inhibited by ivermectin in C4-2 cells. **(B)** The binding of FOXA1 on ARE+FKHD sites or FKHD only sites by ChIP-seq in LNCaP cells.

**Supplementary Fig. S6. (A)** ChIP–qPCR analysis for FOXA1 or AR occupancy, and FAIRE–qPCR analysis of chromatin accessibility at target regulated by AR and FOXA1 in C4-2 cells treated with ivermectin. **(B)** ChIP–qPCR analysis for FOXA1 and FAIRE-PCR analysis of chromatin accessibility at target regulated by FOXA1 in C4-2 cells treated with ivermectin. **(C)** The FOXA1 knockdown reversed ivermectin induced arrest cell cycle at G0/G1 in C4-2 cells treated with ivermectin for 48 hours. **(D)** Western blot analysis of FOXA1, PARP and γH2A.X in FOXA1 siRNA transfected C4-2 cells after ivermectin treatment for 48 hours. **(E)** ITDRF experiments to determine the potency of ivermectin and FOXA1 engagement in LNCaP and 22RV1 cells.

**Supplementary Fig. S7. (A)** Verification of TPP-TR by western blot in 22RV1 cells. **(B and C)** ITDRF experiments to determine the potency of ivermectin and Ku70/Ku80 engagement in LNCaP **(B)** and 22RV1 **(C)** cells.

**Supplementary Fig. S8. (A)** RT-qPCR analysis of BRCA1 and Rad51 in C4-2 and 22RV1 cells treated with ivermectin for 48 hours. **(B)** Western blot analysis of Ku80, Rad51 and PARP and in C4-2 cells after 12 μM ivermectin treatment with or without 1 nM R1881. **(C)** Western blot analysis of Ku80, PARP and γH2A.X in Ku80 siRNA transfected C4-2 cells after ivermectin treatment for 48 hours. **(D)** Western blots showing thermostable Ku70 following indicated heat shocks in the presence (+) or absence (−) of 50 μM ivermectin in DU145 cells. **(E)** Western blot analysis of Ku70, Rad51, γH2A.X and PARP in nuclear and cytoplasmic fractions of DU145 cells. Lamin B and GAPDH was probed as nuclear and cytoplasmic loading control, respectively.

**Supplementary Fig. S1**


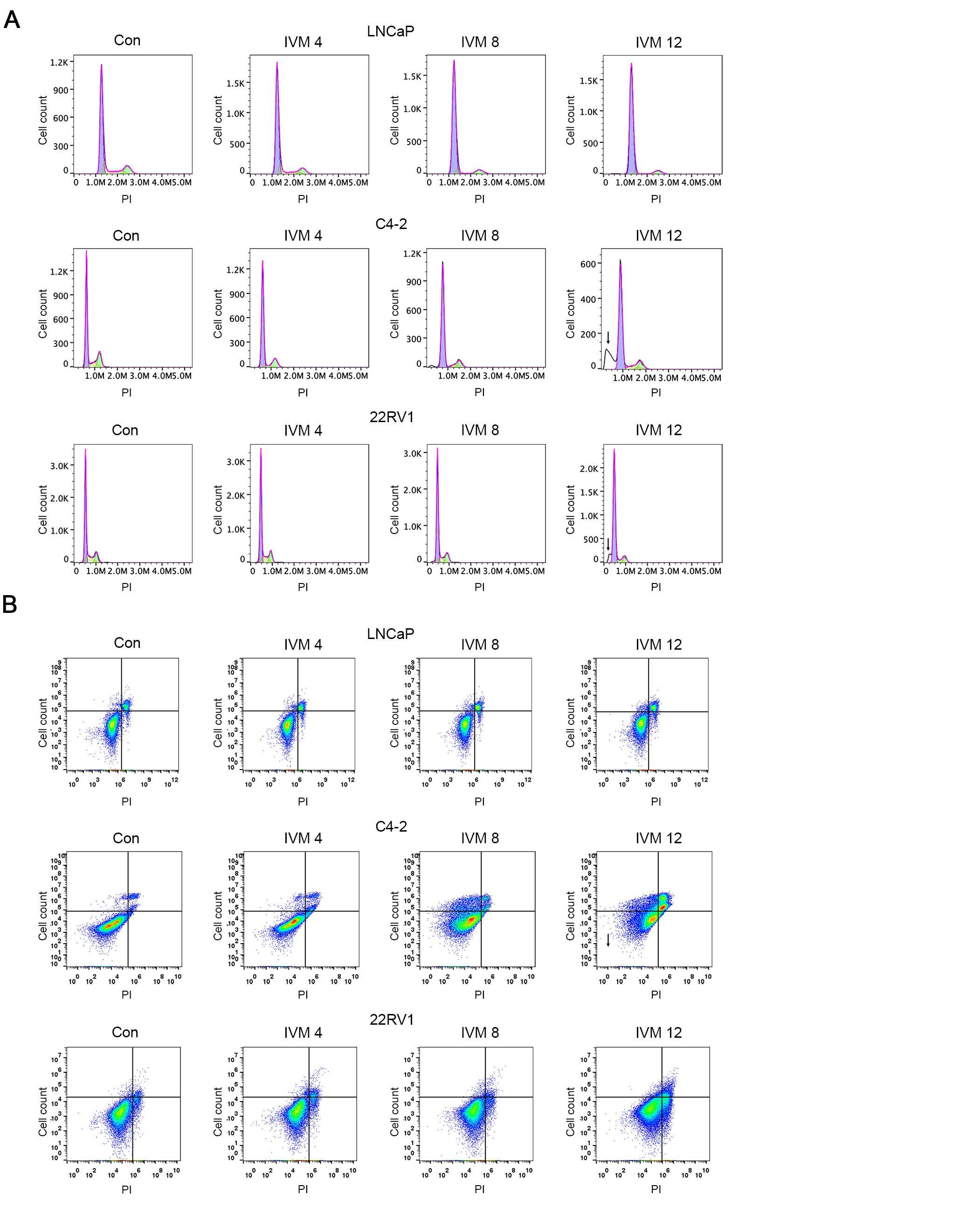


**Supplementary Fig. S2**


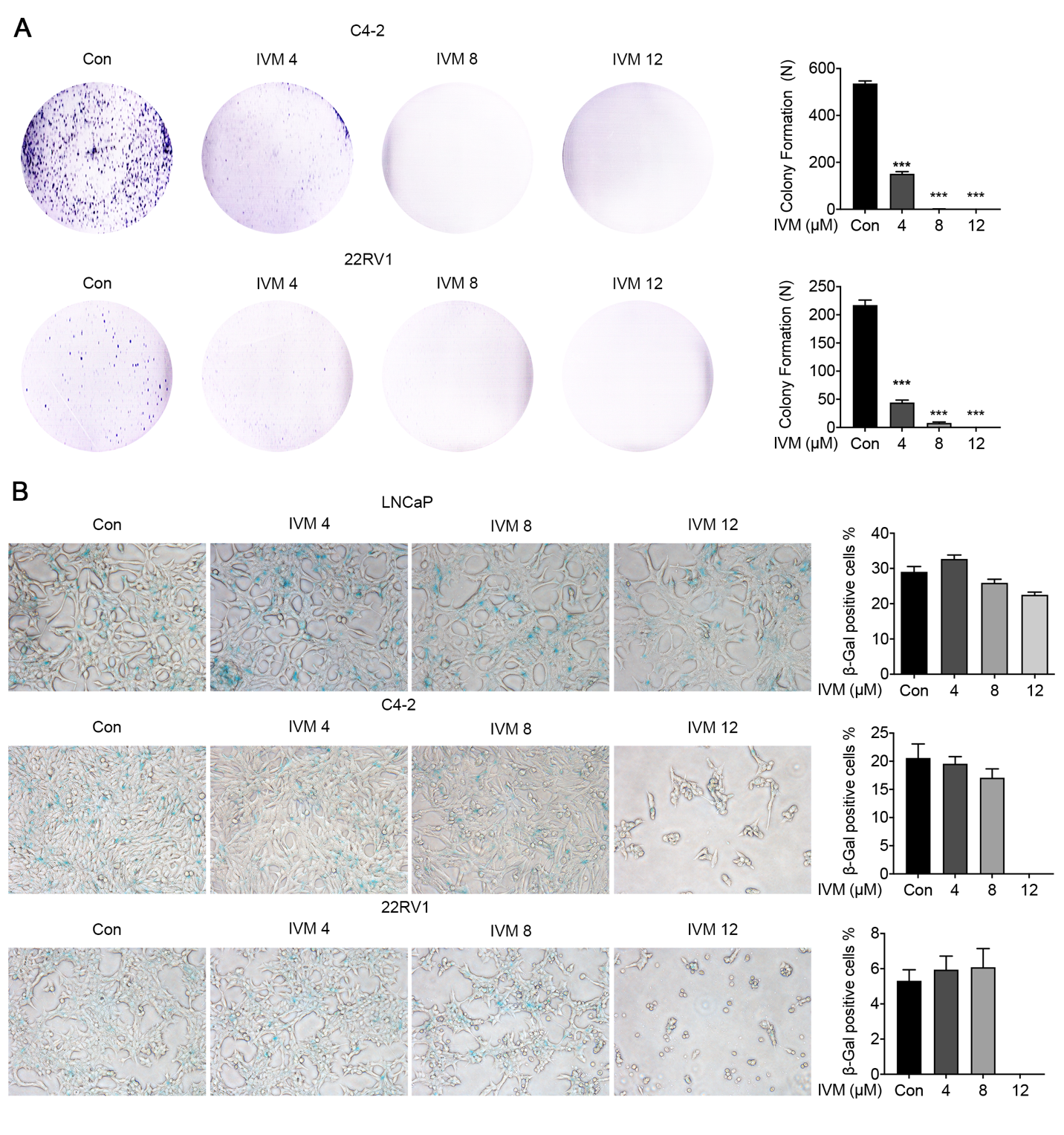


**Supplementary Fig. S3**


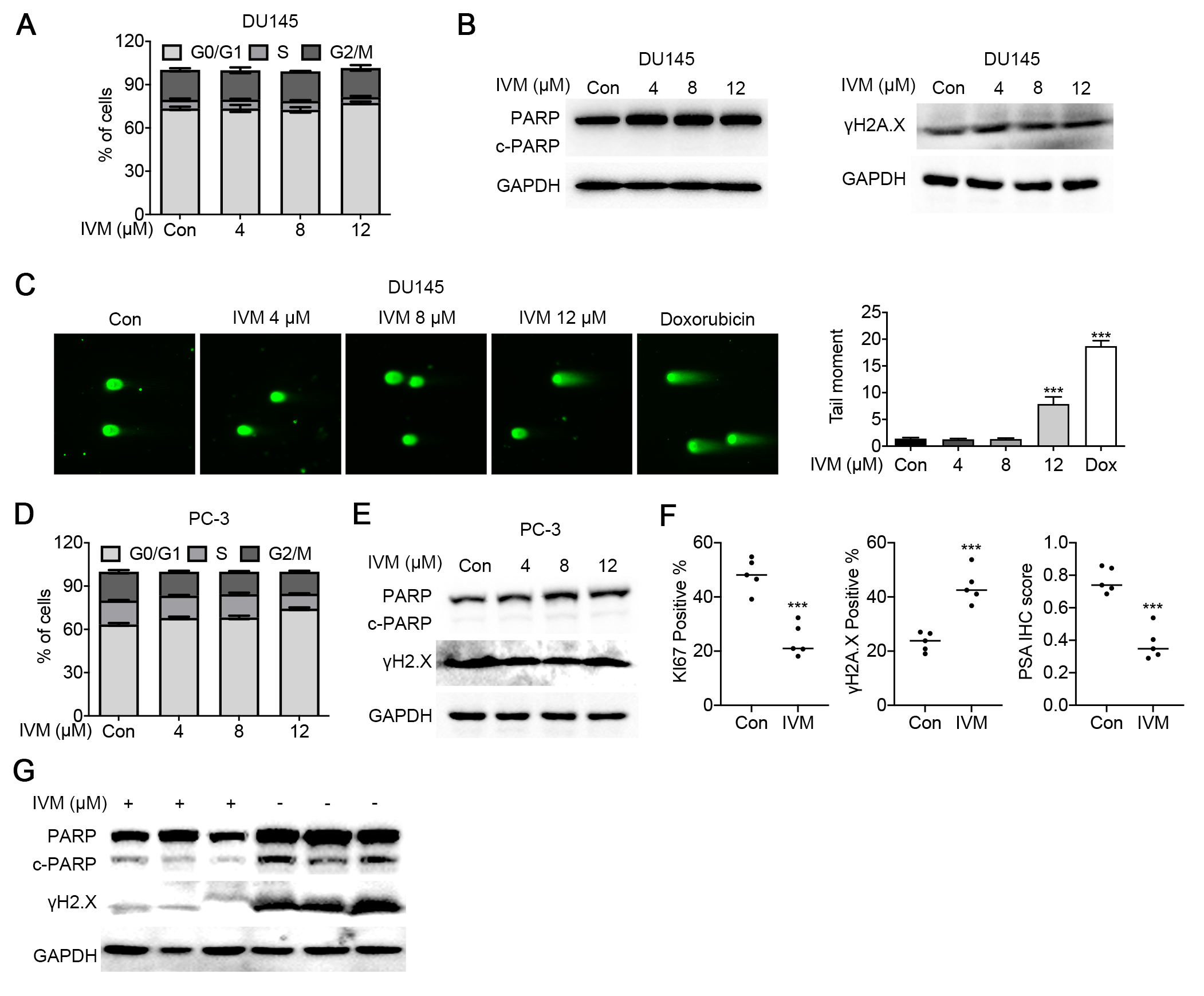


**Supplementary Fig. S4**


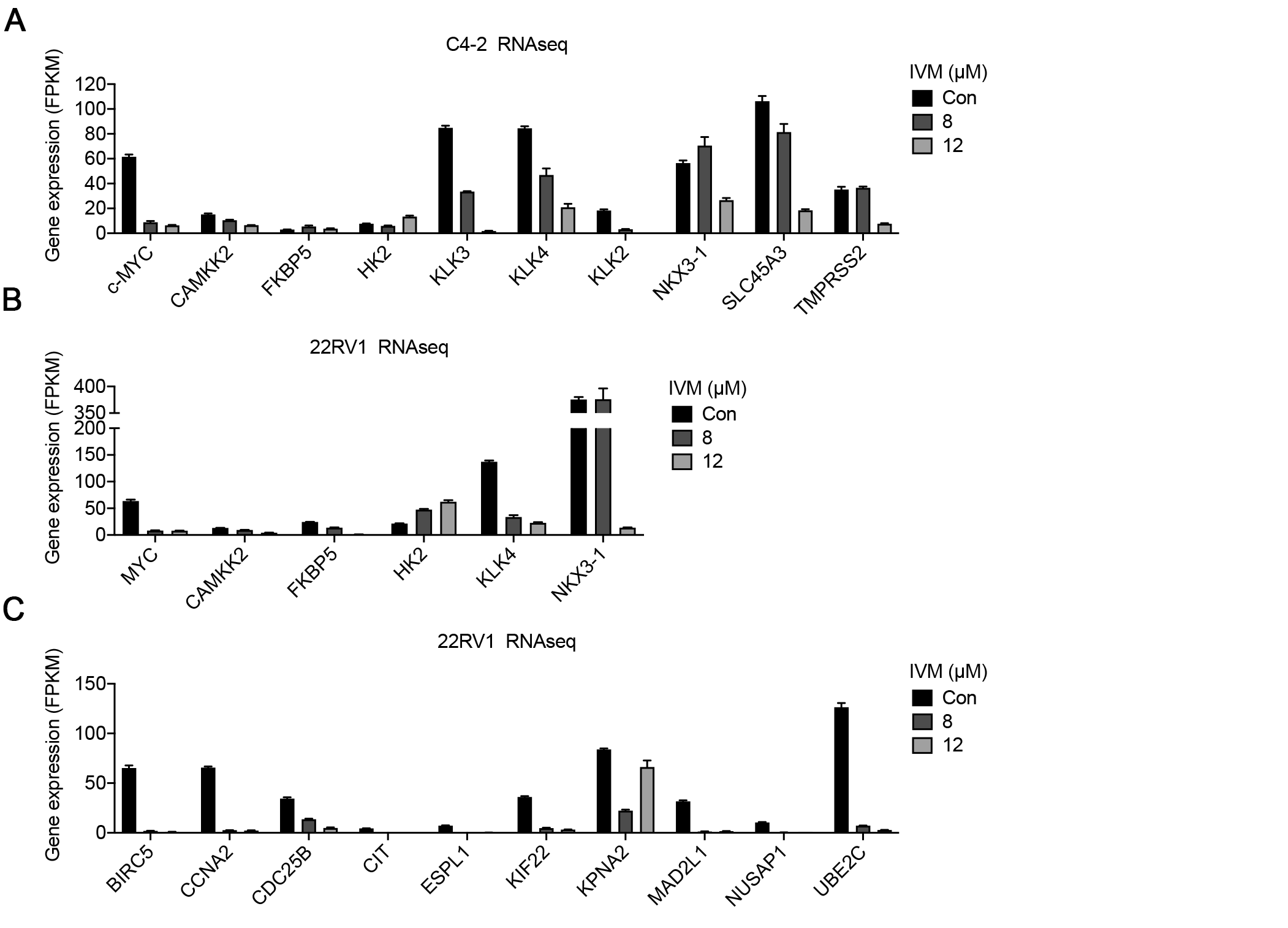


**Supplementary Fig. S5**


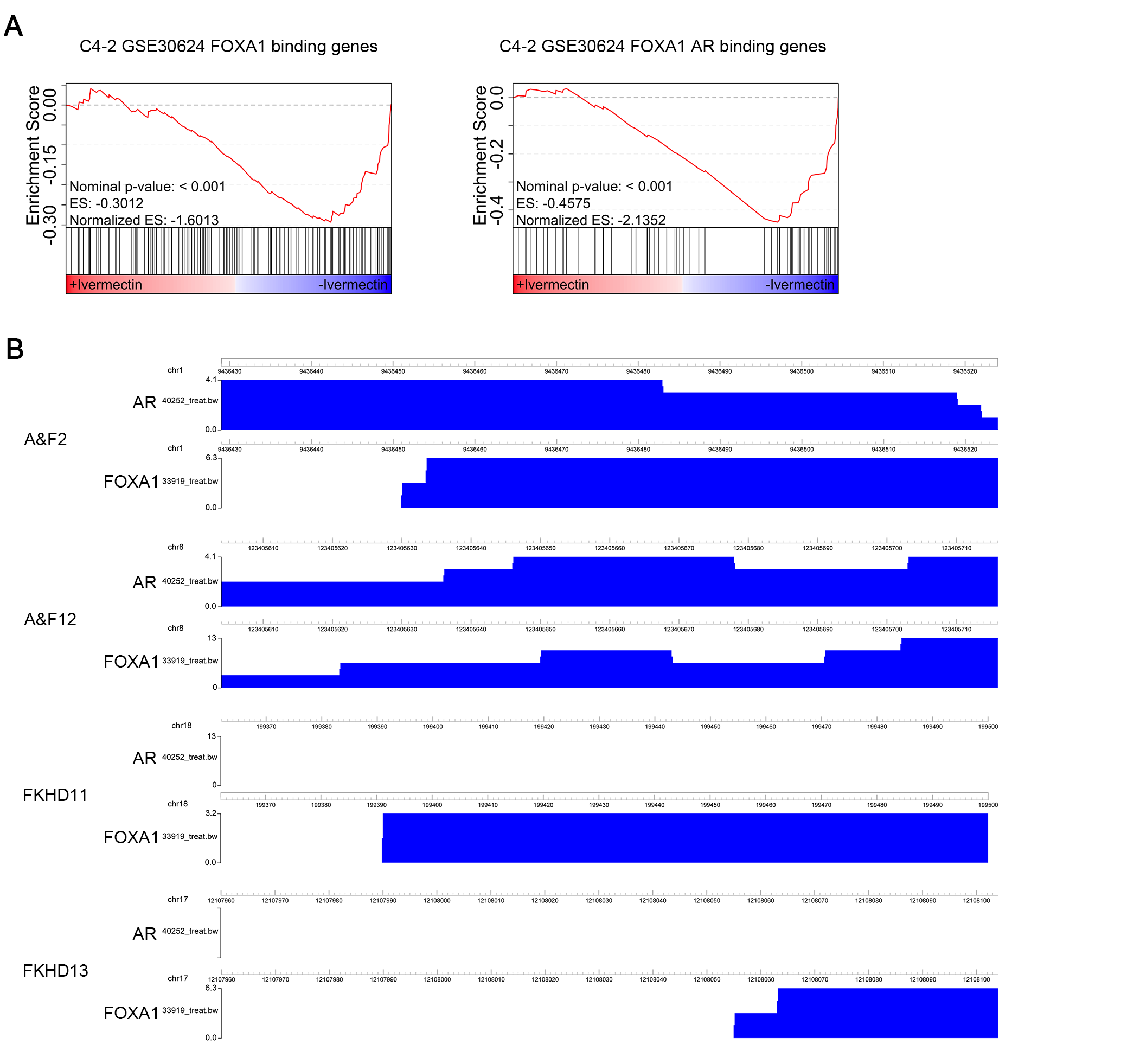


**Supplementary Fig. S6**


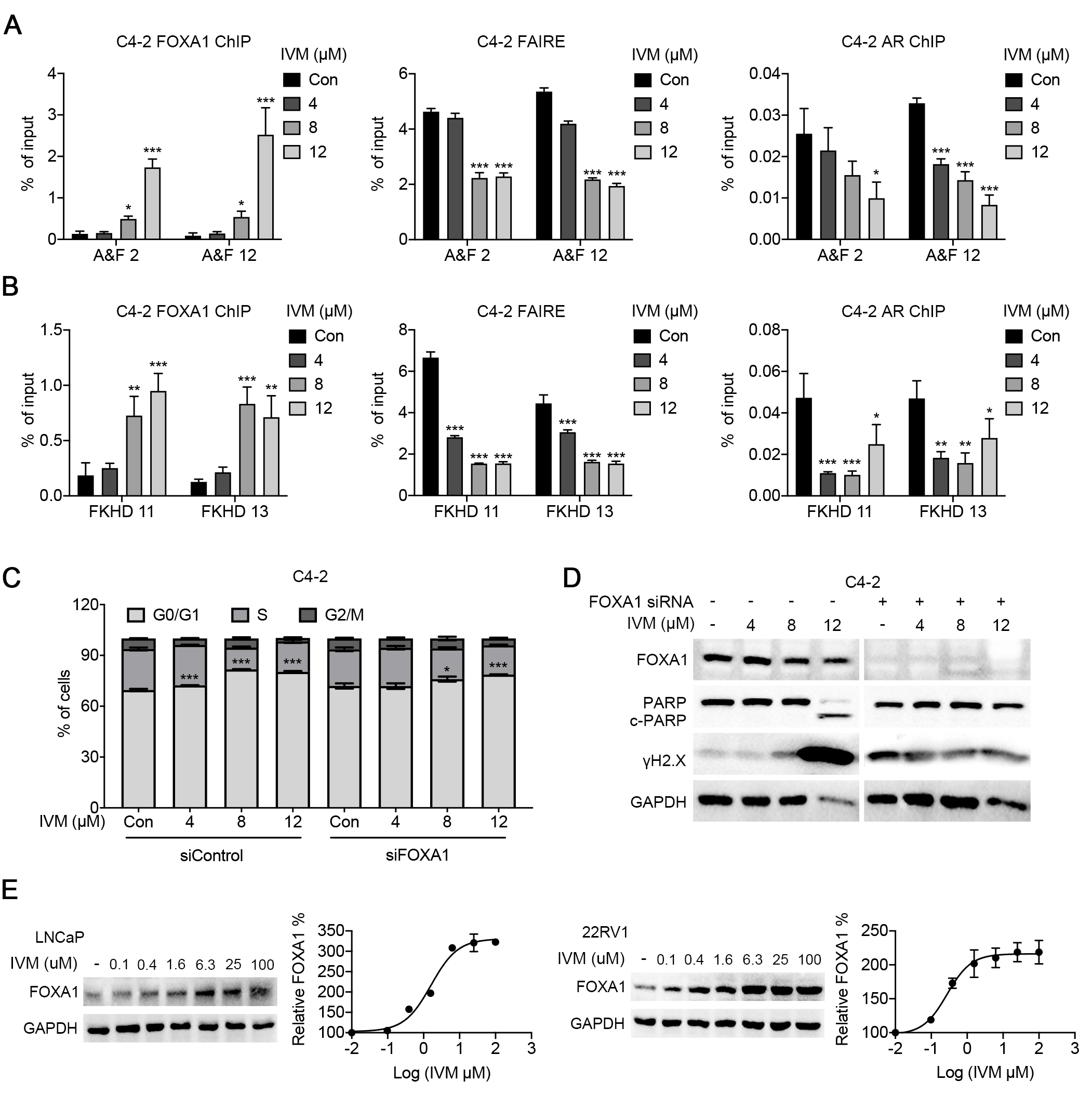


**Supplementary Fig. S7**

**
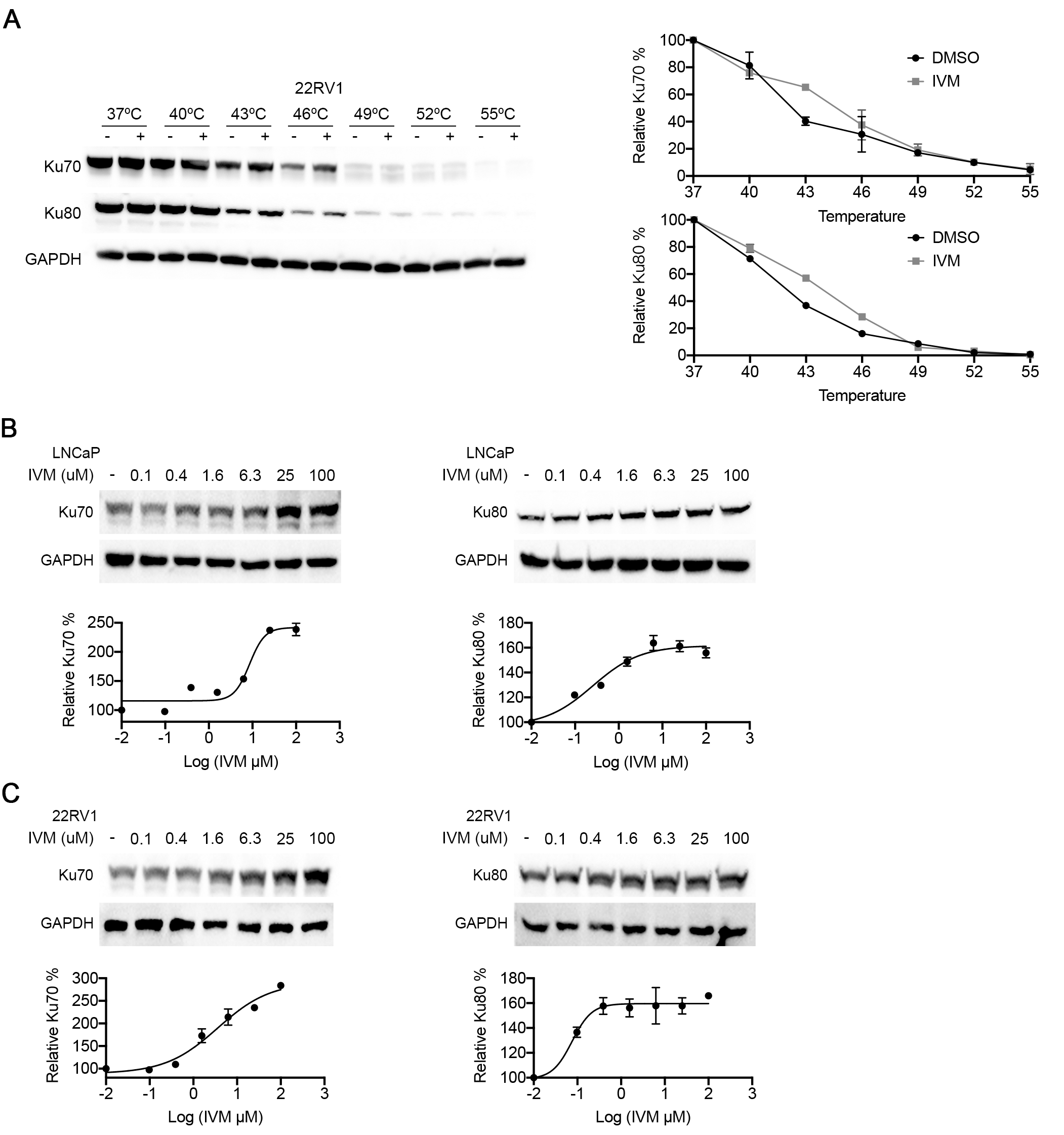
**

**Supplementary Fig. S8**


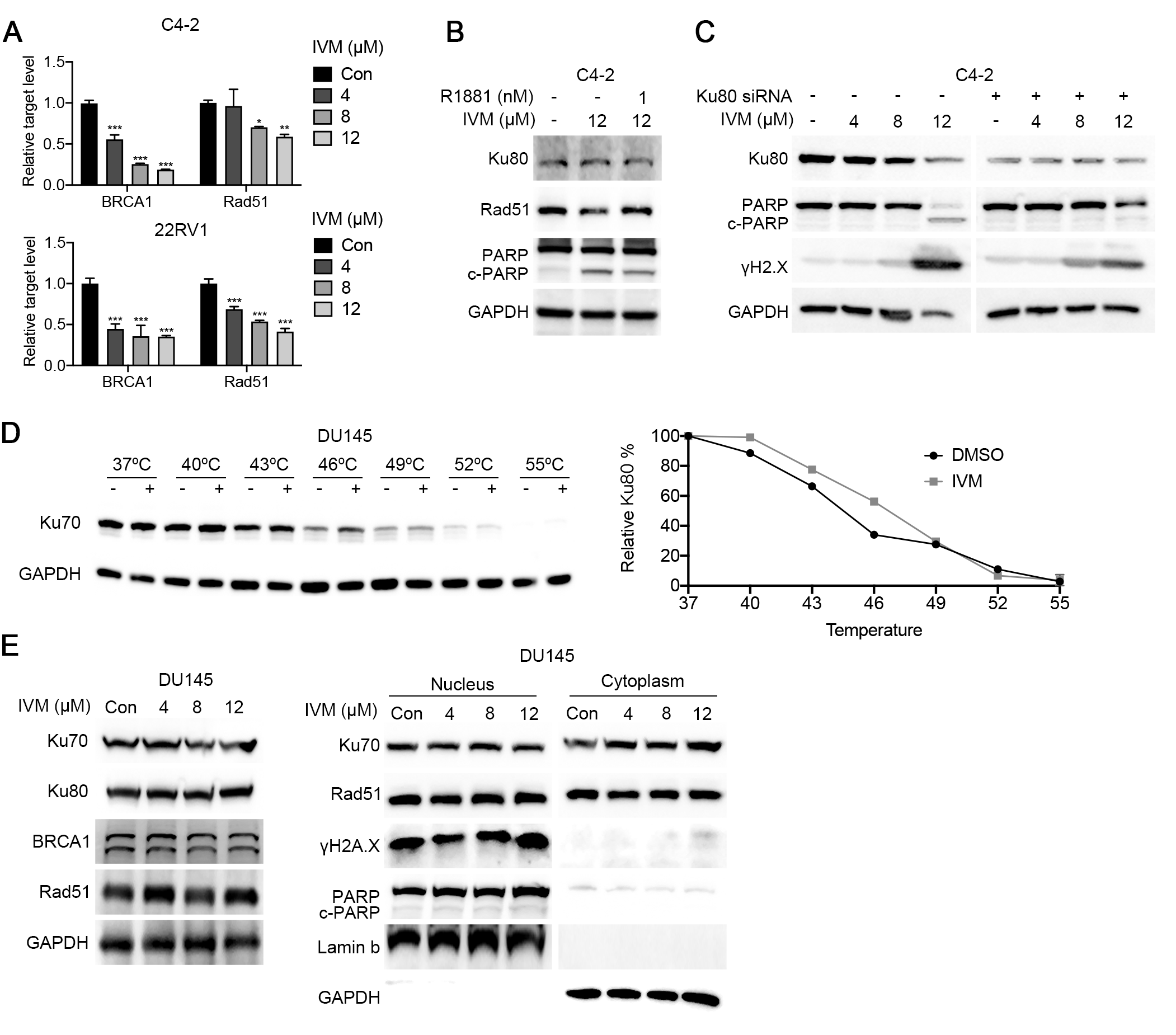


| Supplementary Table S1  **Primer sequence used in RT-qPCR analysis** | | |
| --- | --- | --- |
| Name | Forward Primer | Reverse Primer |
| KLK3 | CAGGTGTAGACCAGAGTGTTTC | CTGTGTCCTCAGAGAAATTGAGT |
| NKX3-1 | TCTGACAGGTGAATTGGATGG | GATTGGAGCAGGGTTTGTTATG |
| TMPRSS2 | TGCTCCAACTCTGGGATAGA | GGATGAAGTTTGGTCCGTAGAG |
| UBE2C | AAAGTGGTCTGCCCTGTATG | GGGACTATCAATGTTGGGTTCT |
| CDC20 | AAGACCTGCCGTTACATTCC | ACATTCCCAGAACTCCAATCC |
| GAPDH | CTCCTCACAGTTGCCATGTA | GTTGAGCACAGGGTACTTTATTG |
| BRCA1 | CAGTCGGGAAACAAGCATAGA | GCACATTCCTCTTCTGCATTTC |
| RAD51 | GGCAGTGATGTCCTGGATAATG | CGGTGGCACTGTCTACAATAAG |
| E2F1 | CTGAGGCCTGGGTGATTTATT | TCTCCCATCTCATATCCATCCT |
| CDKN3 | TCGGTTTATGTGCTCTTCCA | TTTTGACAGTTCCCCTCTGG |
| CDCA2 | GACAGAGCATGTGCAGTTGAA | TGAGCTCTGAAAGGGGAAGA |
| CAMKK2 | TCTCACCACGTCTCCATCAC | GCCCTTTCCAATTTCATCCT |
| MET | CCGTGAAGATCCCATTGTCTAT | GACCATTCTCGGGACACTAAC |
| MMP7 | GGAGGCATGAGTGAGCTACAG | GGCCAAAGAATTTTTGCATC |
| SOX9 | AGTACCCGCACTTGCACAAC | GTAATCCGGGTGGTCCTTCT |
| FOXA1 | GTATTCCAGACCCGTCCTAAAC | CTGTTGACGGTTTGGTTTGTG |
| KLK3-ehancer | TCGATTGTCCTTGACAGTAAACA | TCTCAGATCCAGGCTTGCTT |
| NKX3-1-enhancer | CTGGCAAAGAGCATCTAGGG | GGCACTTCCTGAGCAAACTT |
| E2F1-enhancer | GGGACACGGCCACATTGT | TGGTCCCCAAGTCCTTCCA |
| MET-enhancer | TGAGACACAGTGGATGTGTGA | GATCTCCCTGGTTGTTGCAT |
| A&F 2 | GGCTTCTTATCATGCCTGGA | AAGAACAGACAGTACGGAGTGG |
| A&F 12 | AGCATGTGTTTGCATGGGTA | CACAGGGAAAGATCACTAAGACC |
| FKHD 11 | TTGCGAGTAAGCCAAAGTCA | GCTGAAACAAGAAGGCCAAG |
| FKHD 13 | TGCTGCTGGAGTTTTGAATG | TTGGCAGTATTTATCGAGACCA |
