# Supplementary material for: Integrated analysis reveals FOXA1 and Ku70/Ku80 as targets of ivermectin in prostate cancer
Source: Cell Death Dis. 2022 Sep 1;13(9):754. doi: 10.1038/s41419-022-05182-0 (PMC9436997; doi:10.1038/s41419-022-05182-0)

Fig. 2C LNCaP

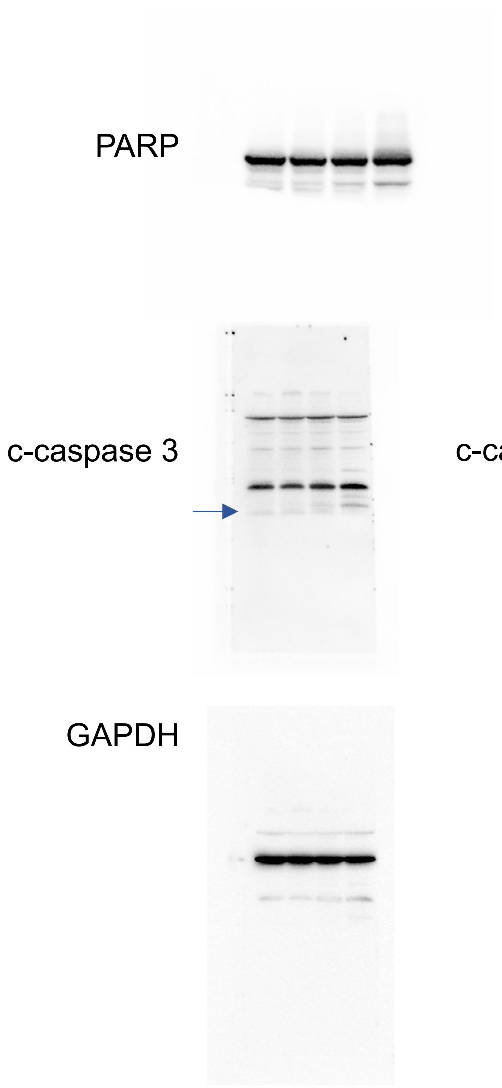

Fig. 2C C4-2

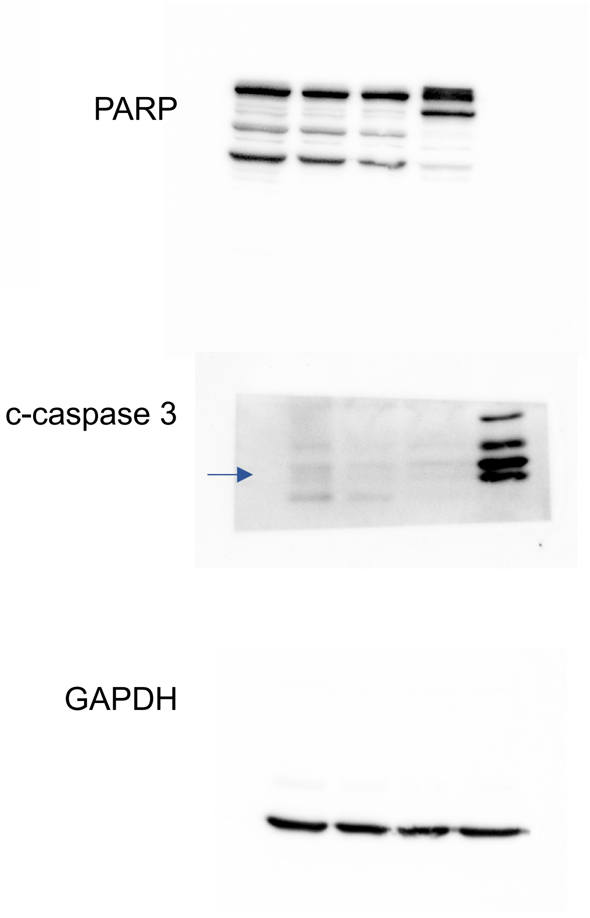

Fig. 2C 22RV1

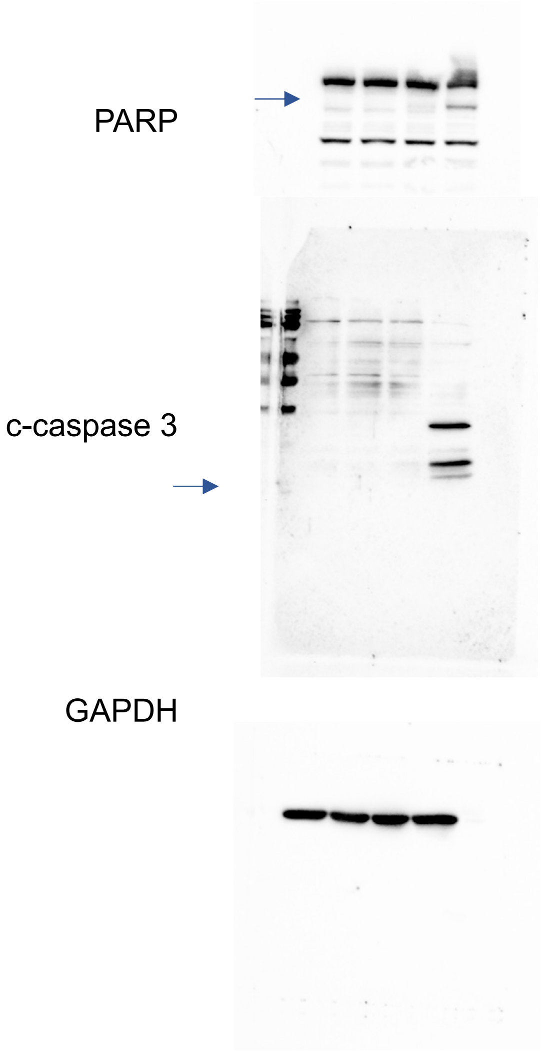

Fig. 2E LNCaP

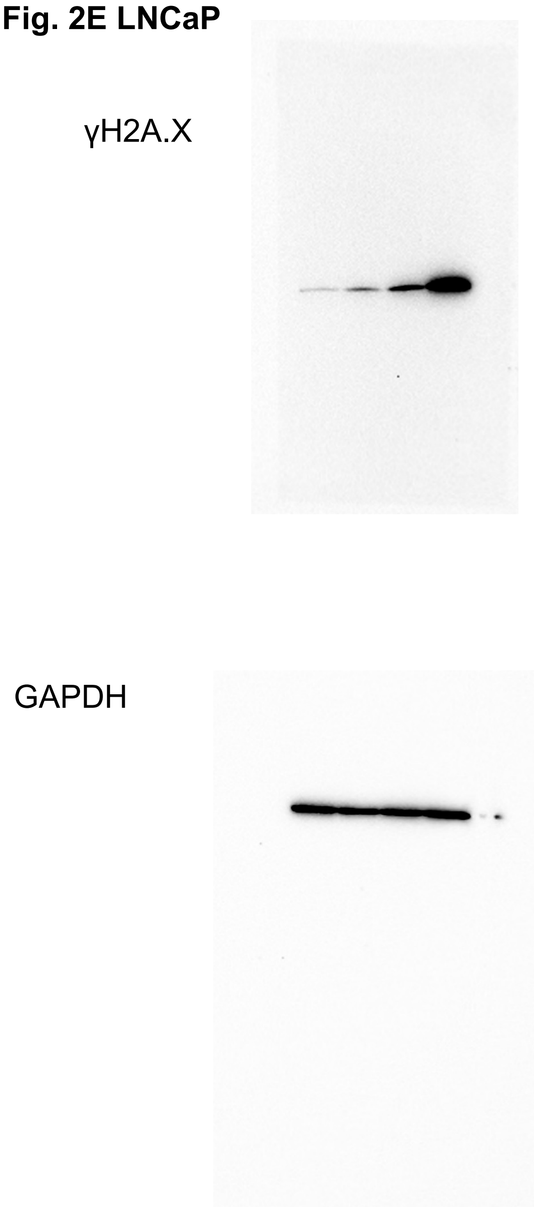

Fig. 2E C4-2

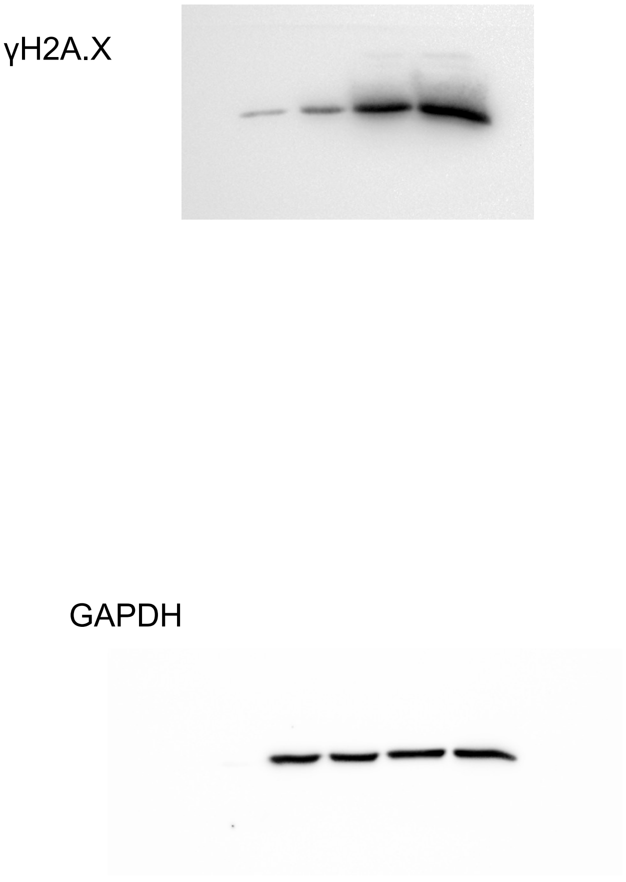

Fig. 2E 22RV1

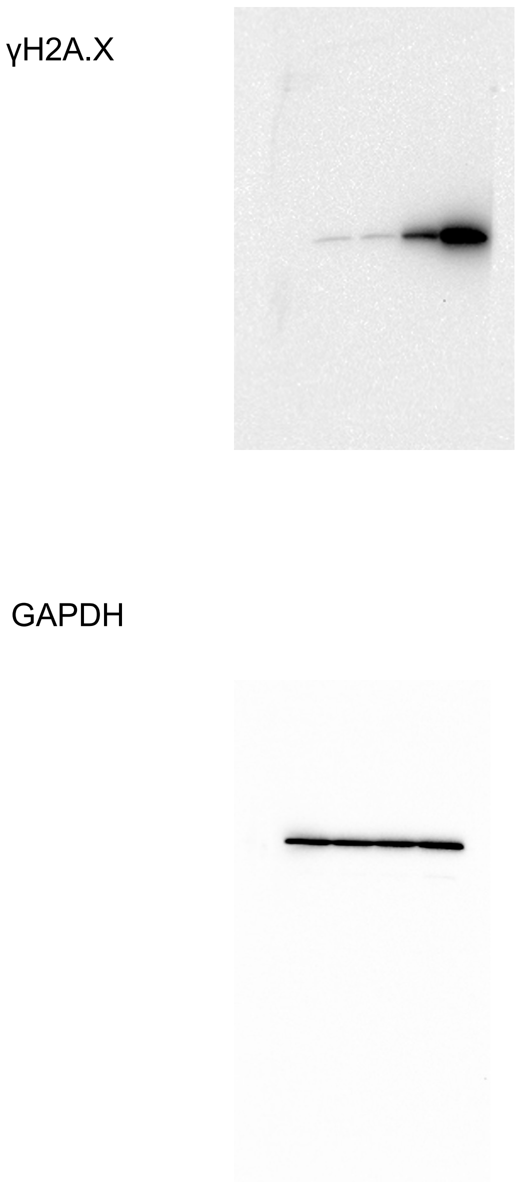

Fig. 3A LNCaP

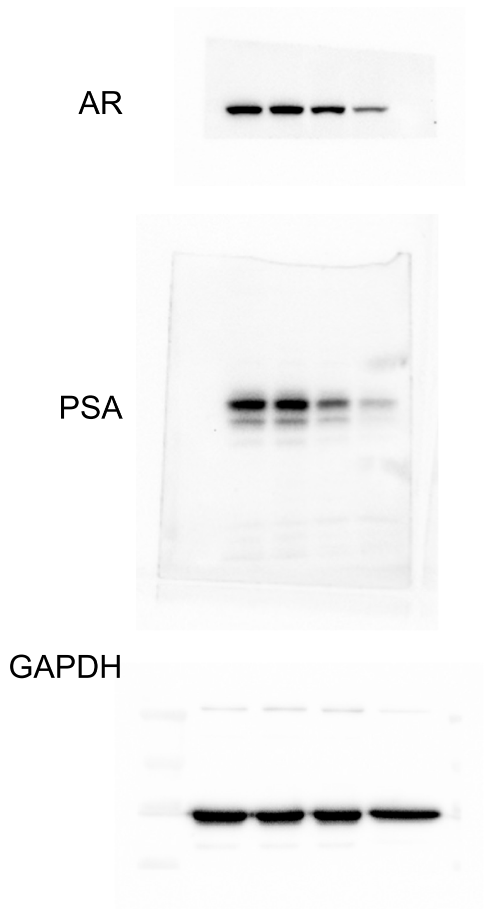

Fig. 3A C4-2

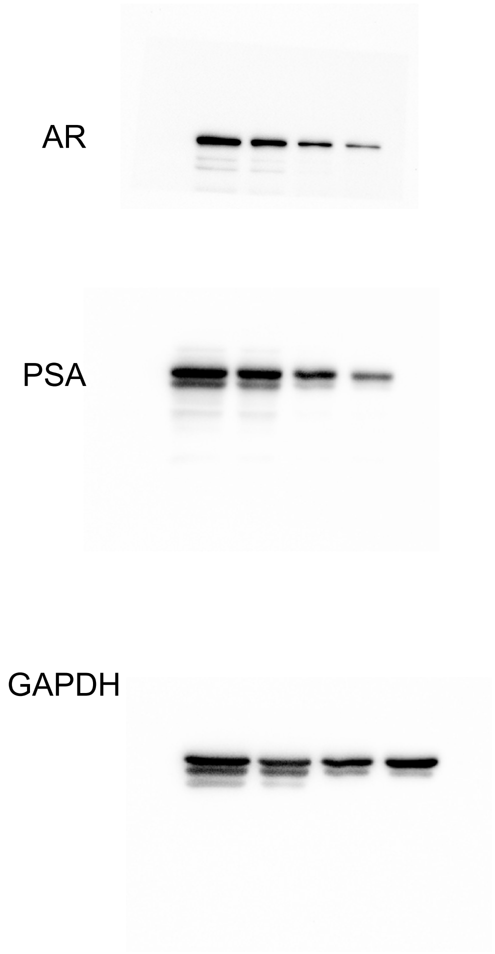

Fig. 3F LNCaP

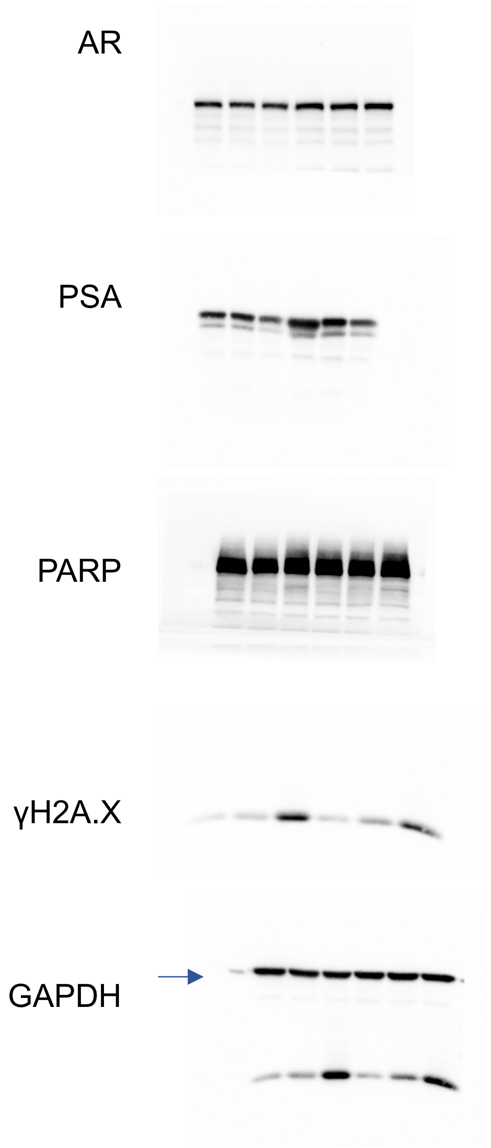

Fig. 3E LN95

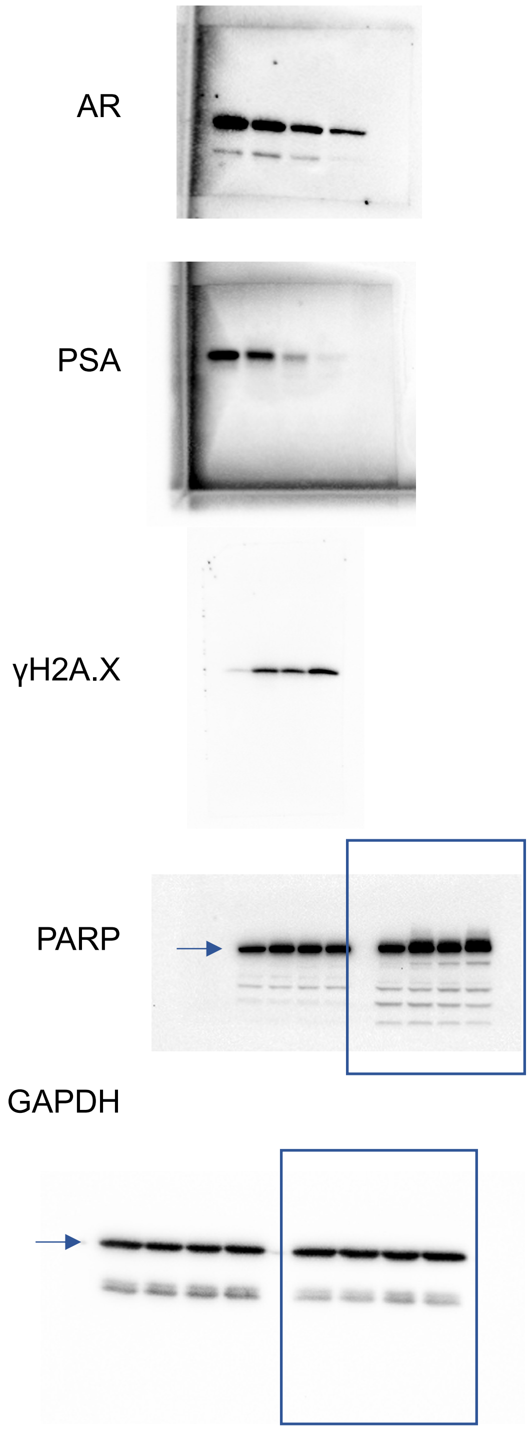

Fig. 3E VCaP

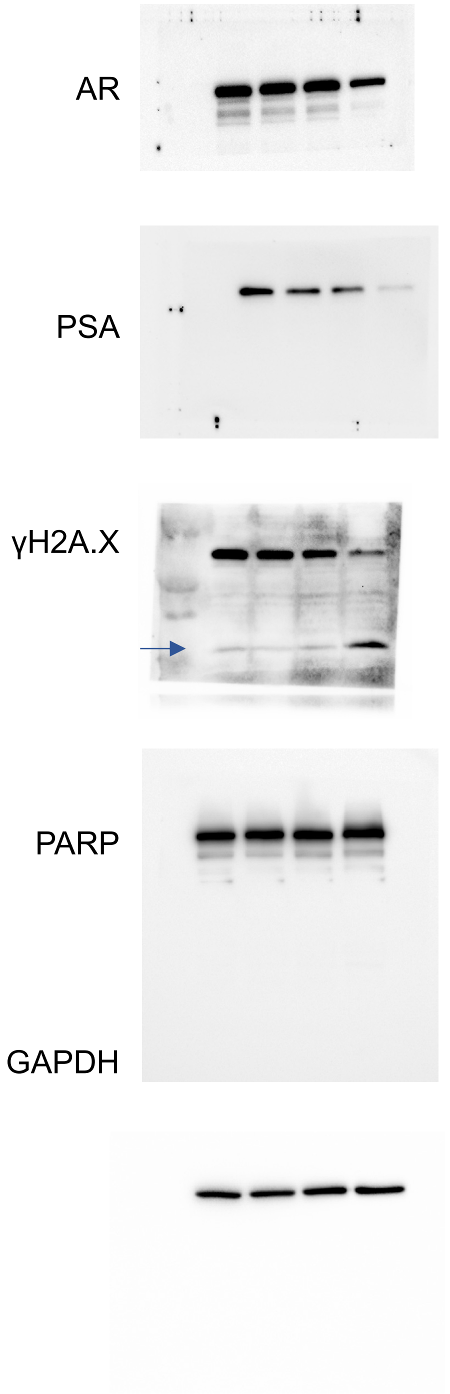

Fig. 3F C4-2

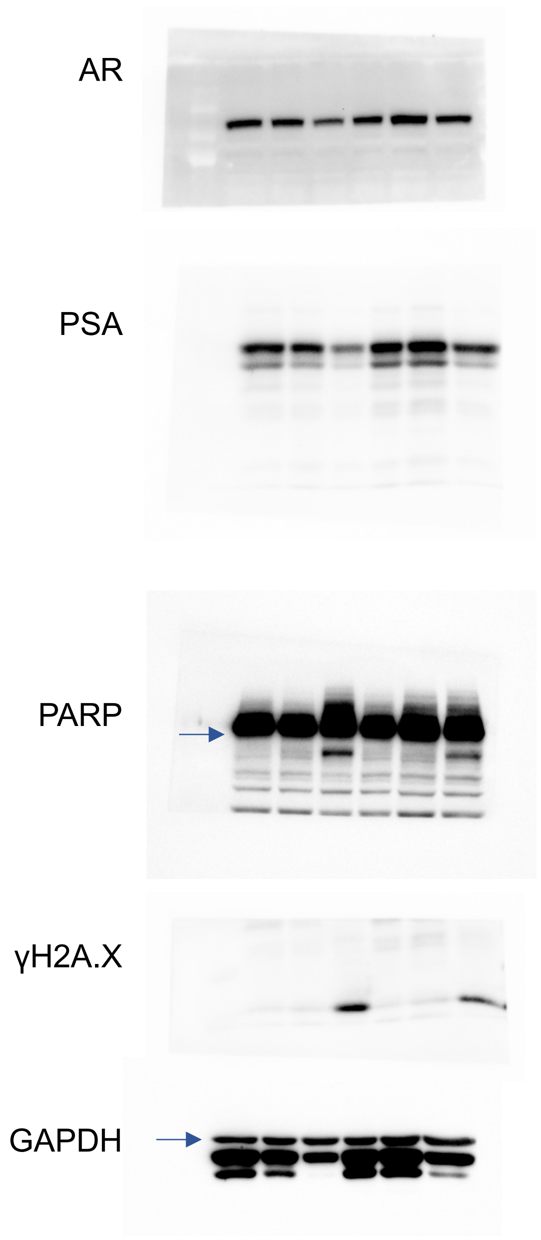

Fig. 4E C4-2

E2F1

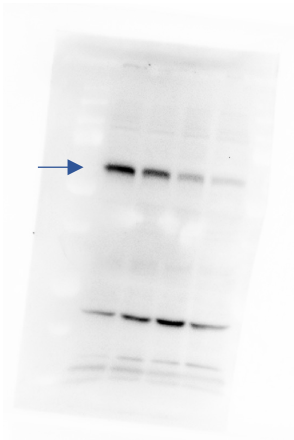

GAPDH

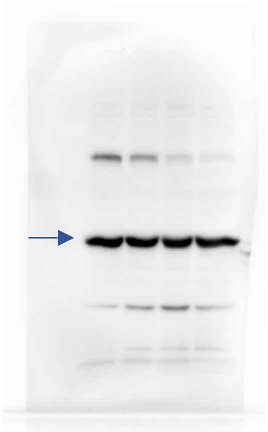

Fig. 4E 22RV1

E2F1

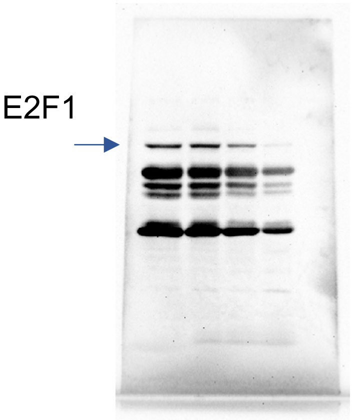

GAPDH

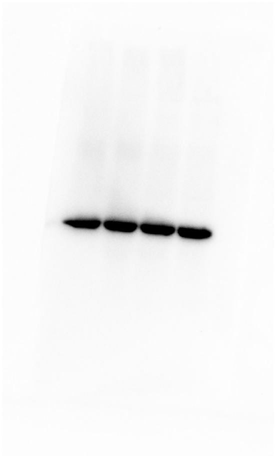

Fig. 4G C4-2

E2F1

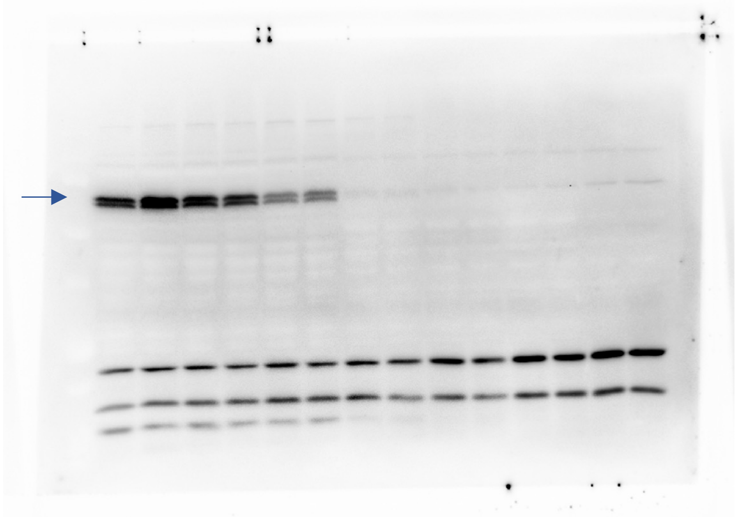

GAPDH

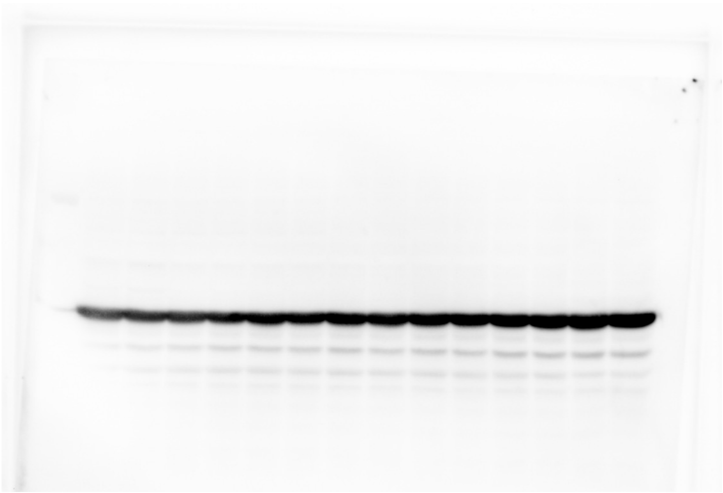

Fig. 5C LNCaP

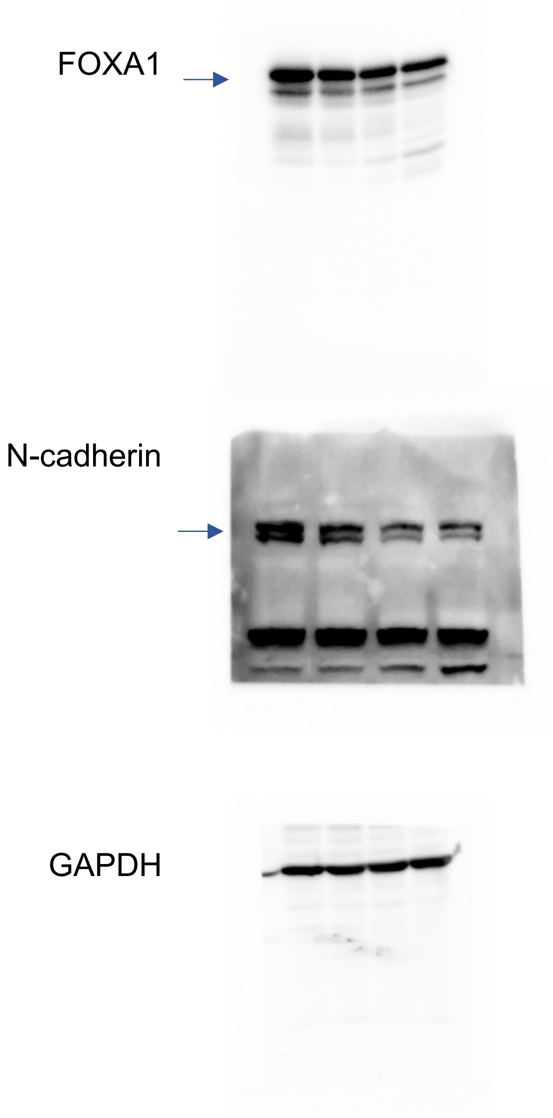

Fig. 5C C4-2

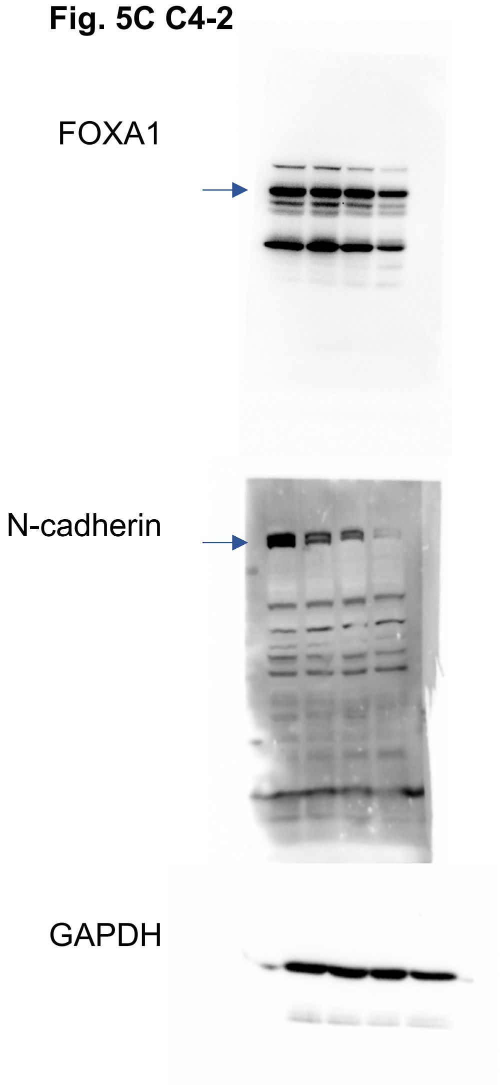

Fig. 5G LNCaP

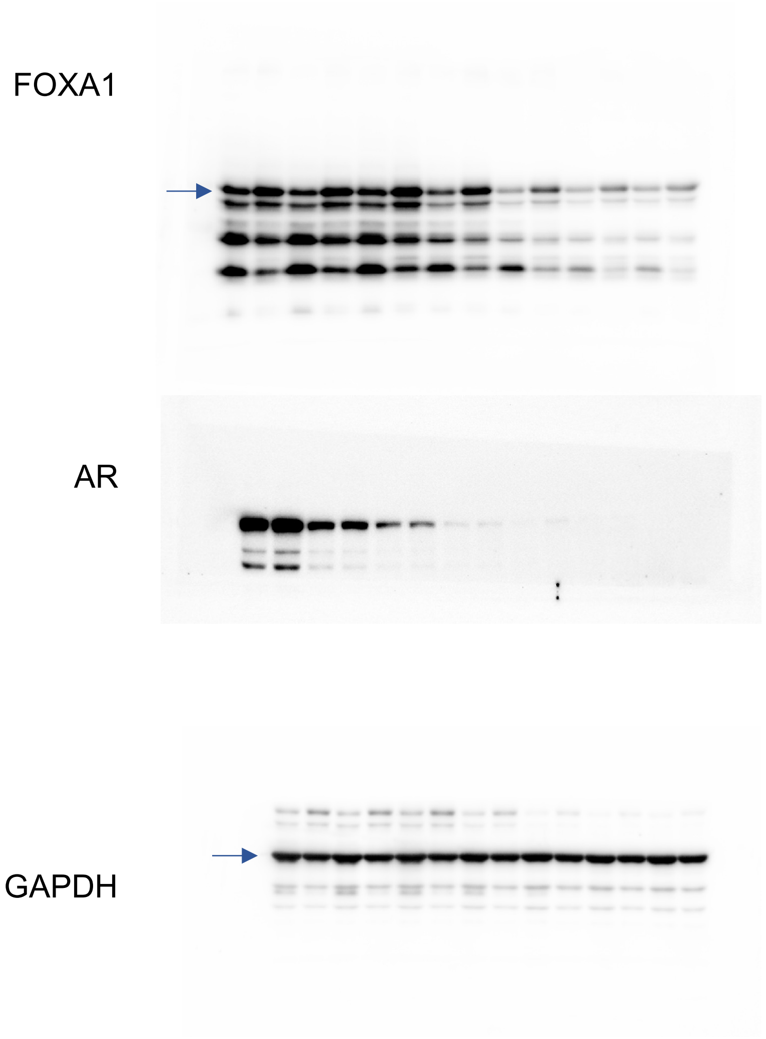

Fig. 5H C4-2

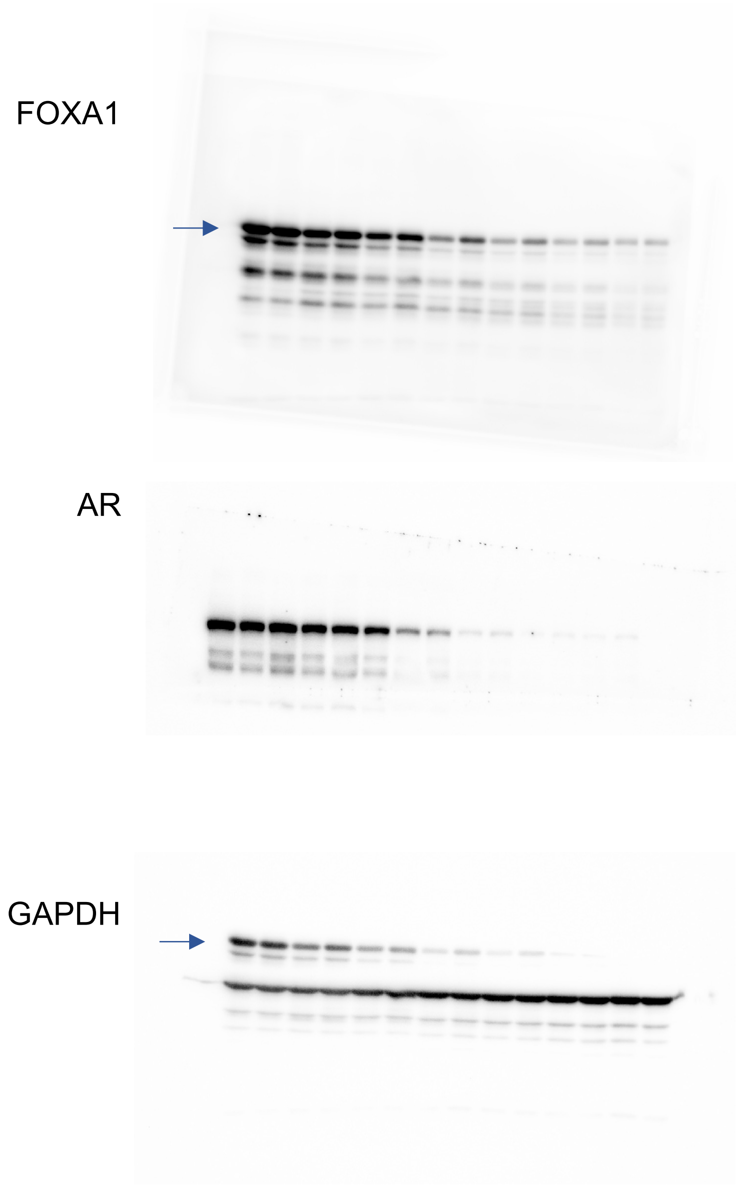

Fig. 5I 22RV1

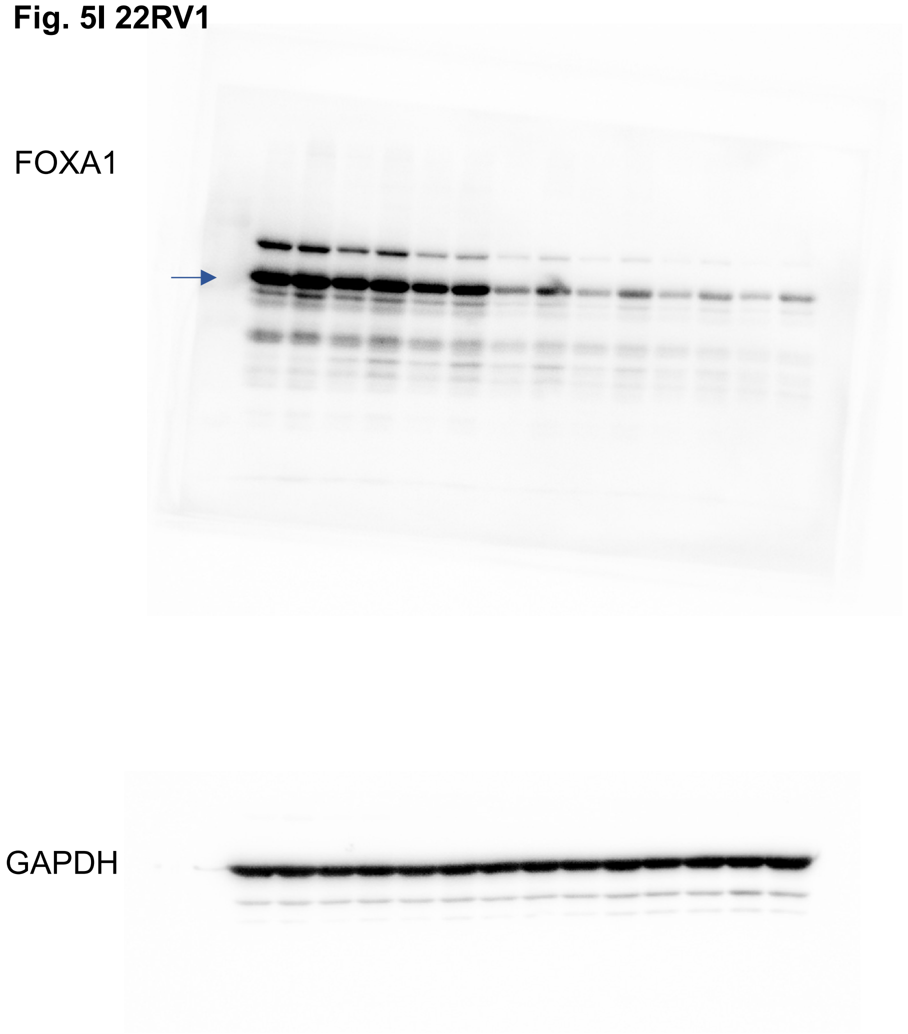

Fig. 6D LNCaP

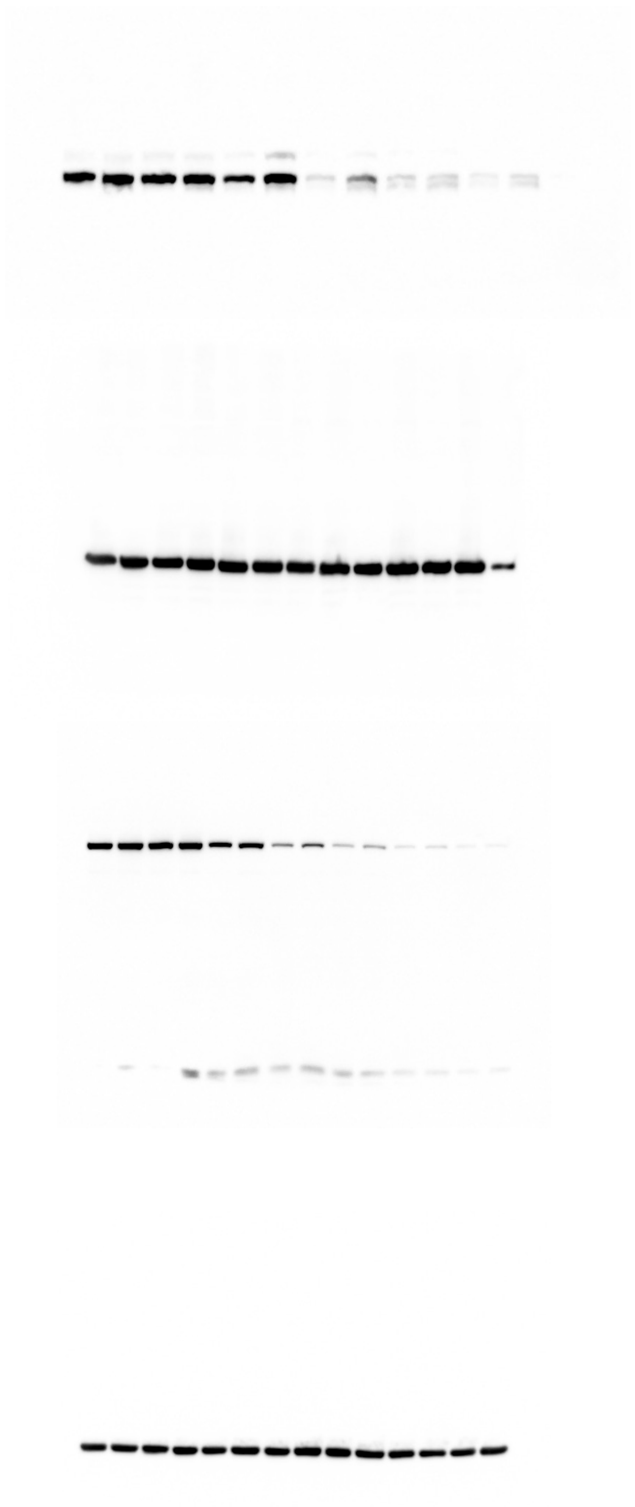

Fig. 6E C4-2

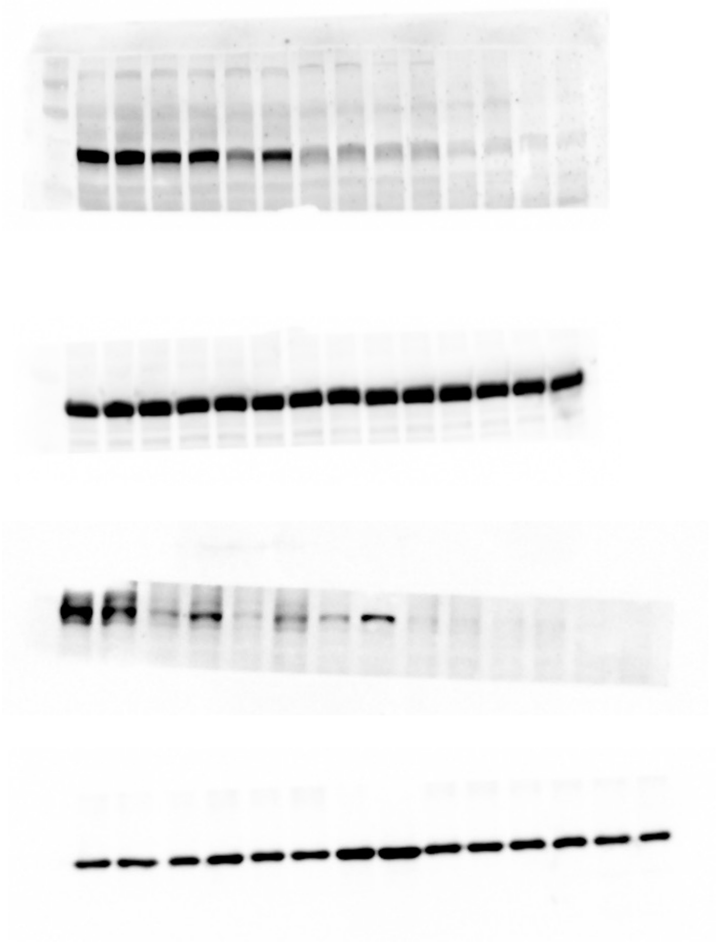

Fig. 7B C4-2

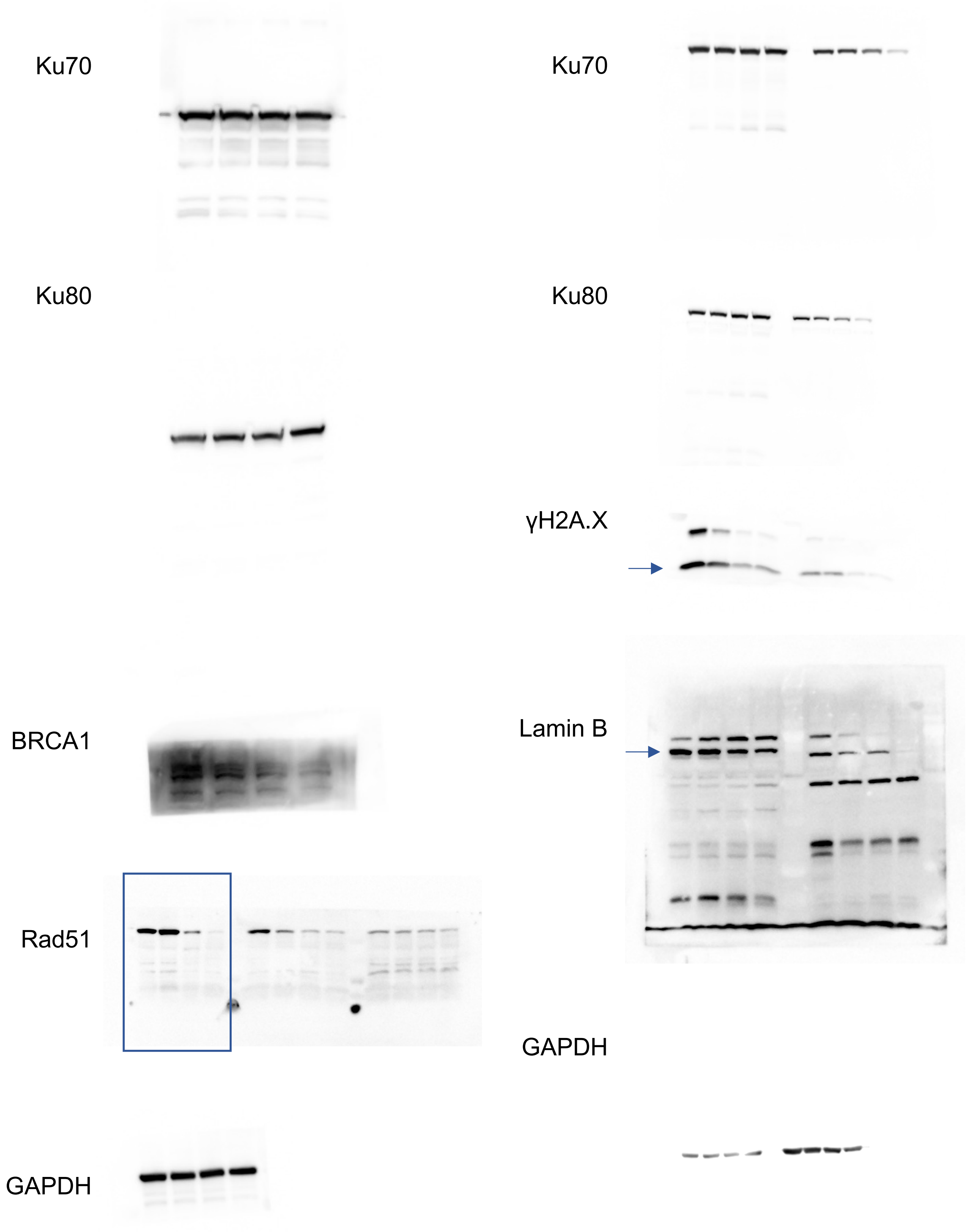

Fig. 7C 22RV1

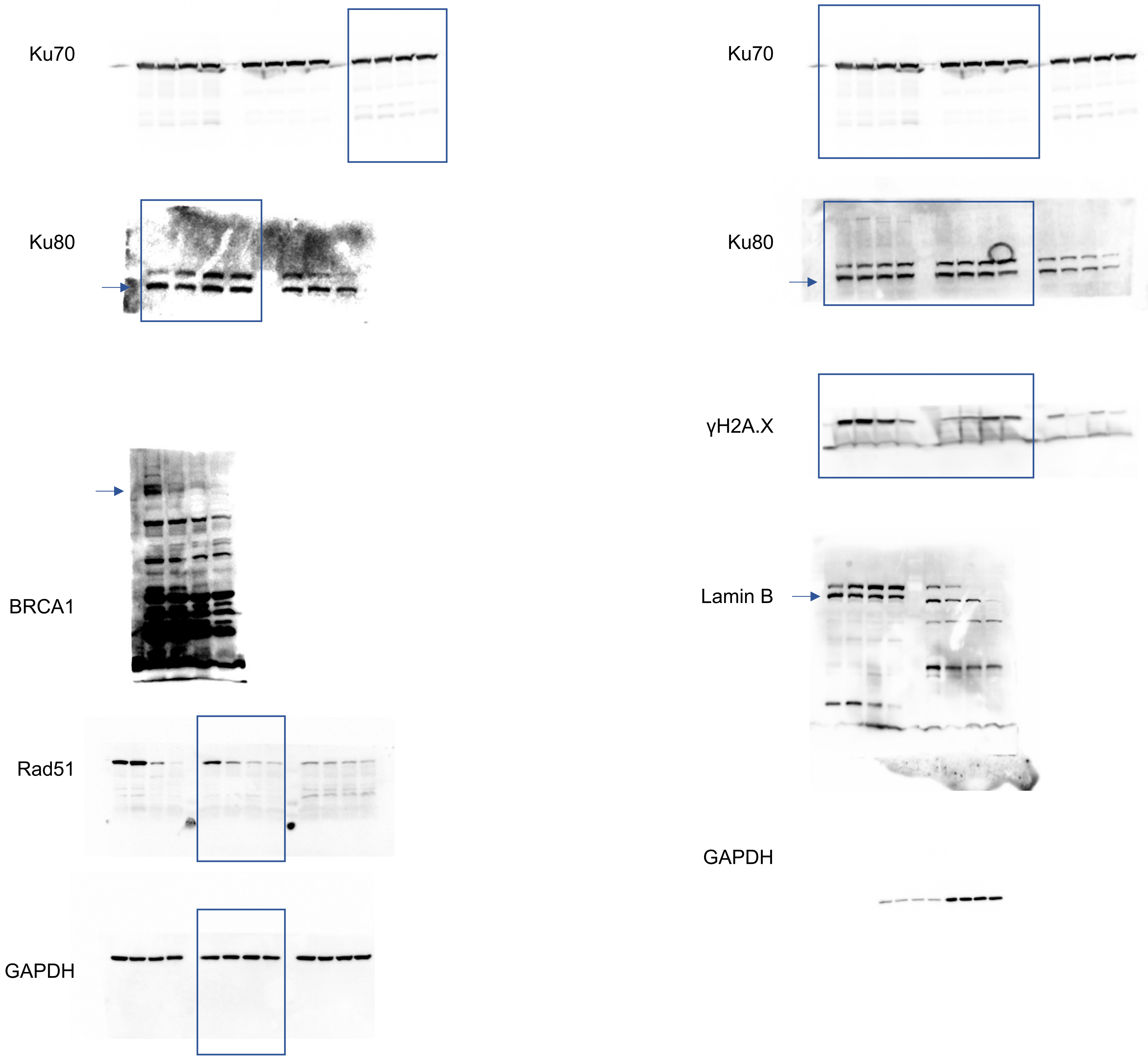

Fig. S3B DU145

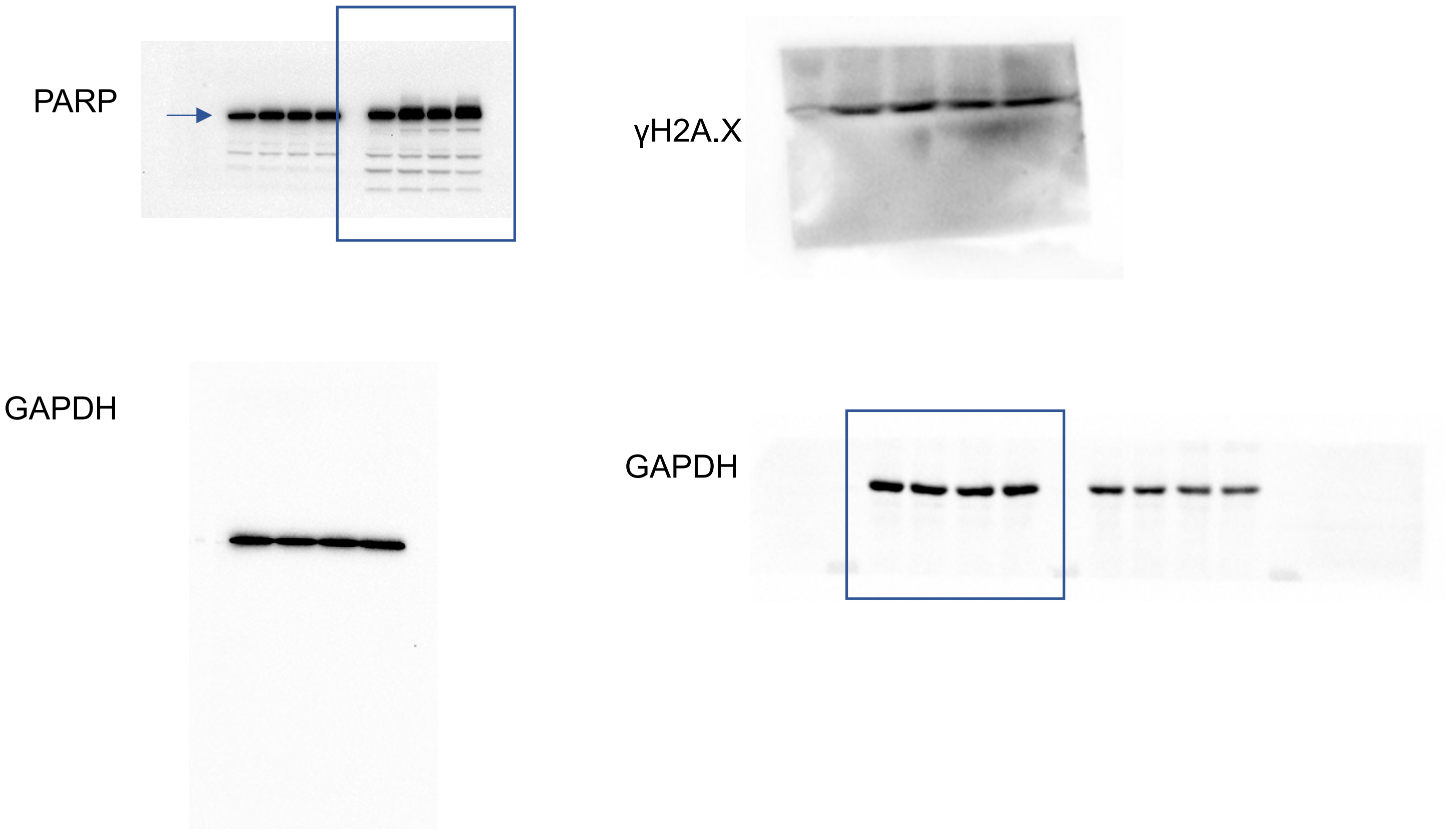

Fig. S3E PC-3

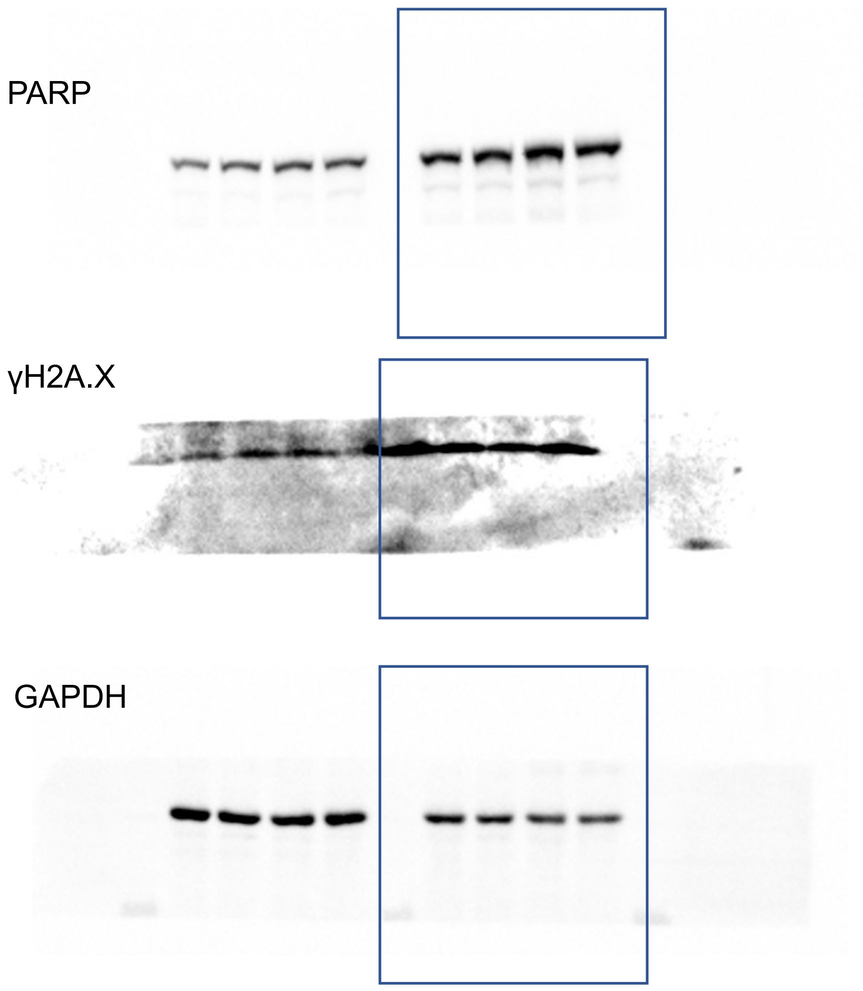

Fig. S3F 22RV1 xenograft model

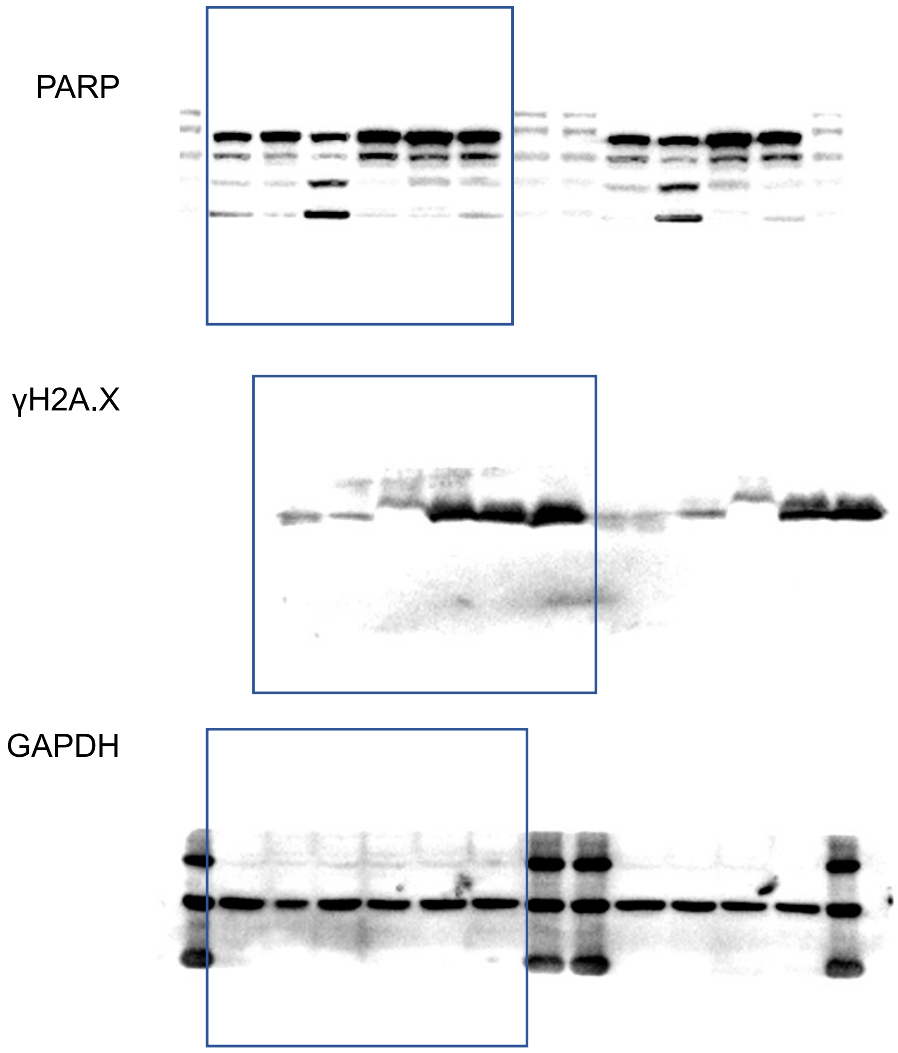

Fig. S6D C4-2

FOXA1

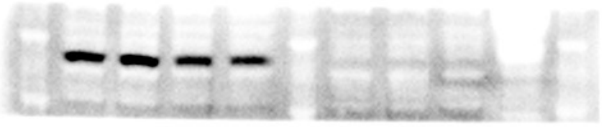

PARP

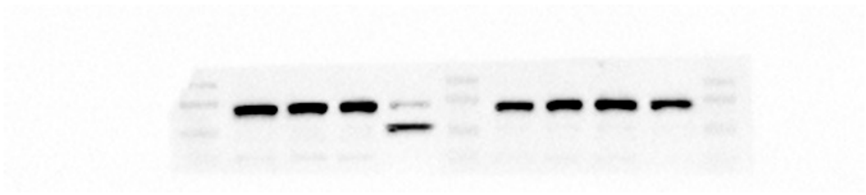

γH2A.X

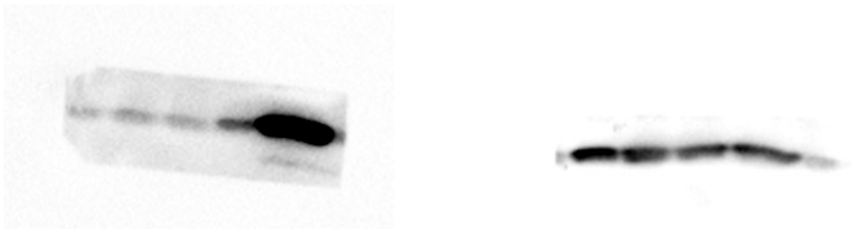

GAPDH

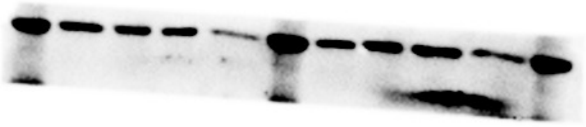

Fig. S6E LNCaP

FOXA1

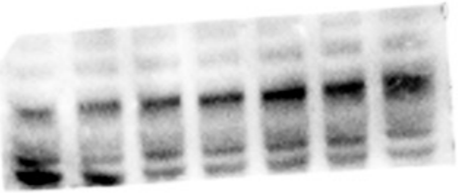

GAPDH

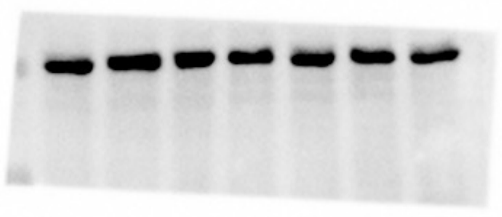

Fig. S6E 22RV1

FOXA1

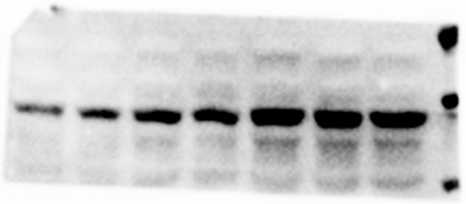

GAPDH

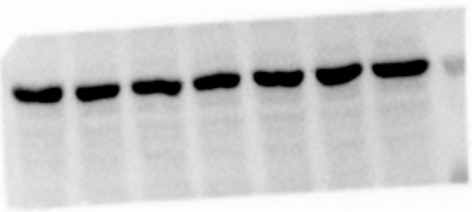

Fig. S7A 22RV1

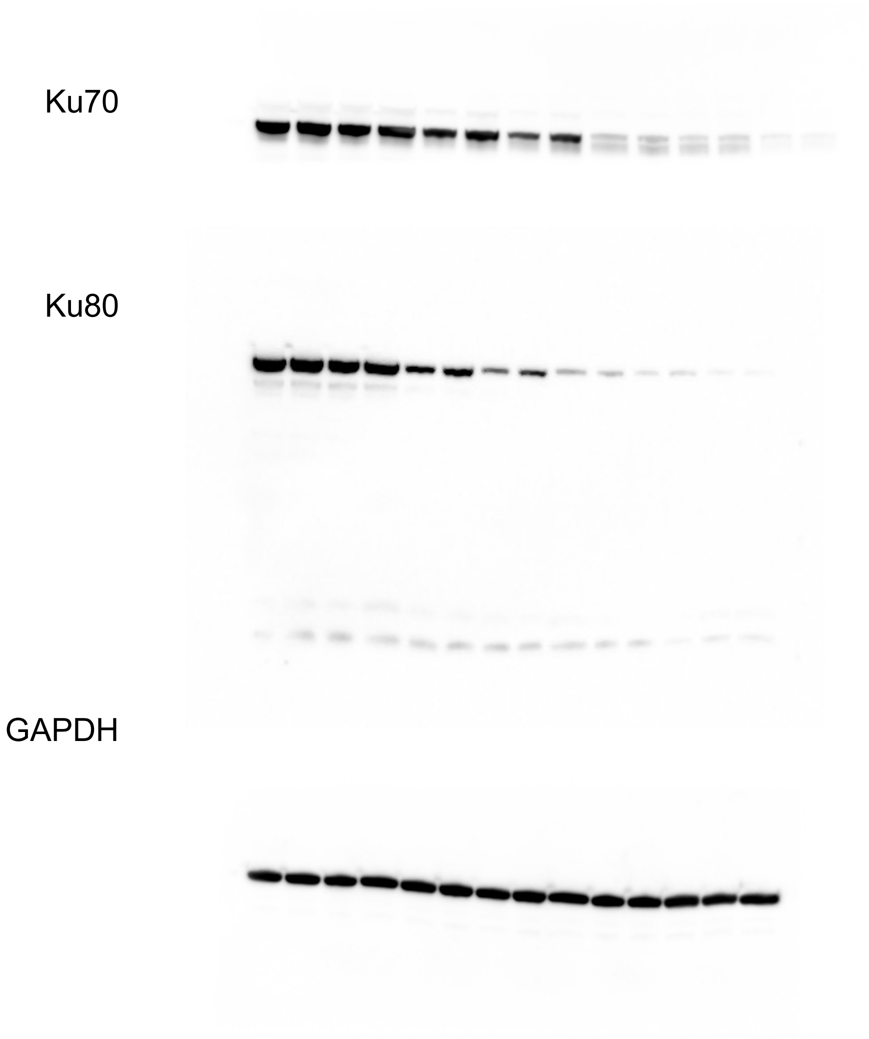

Fig. S7B LNCaP

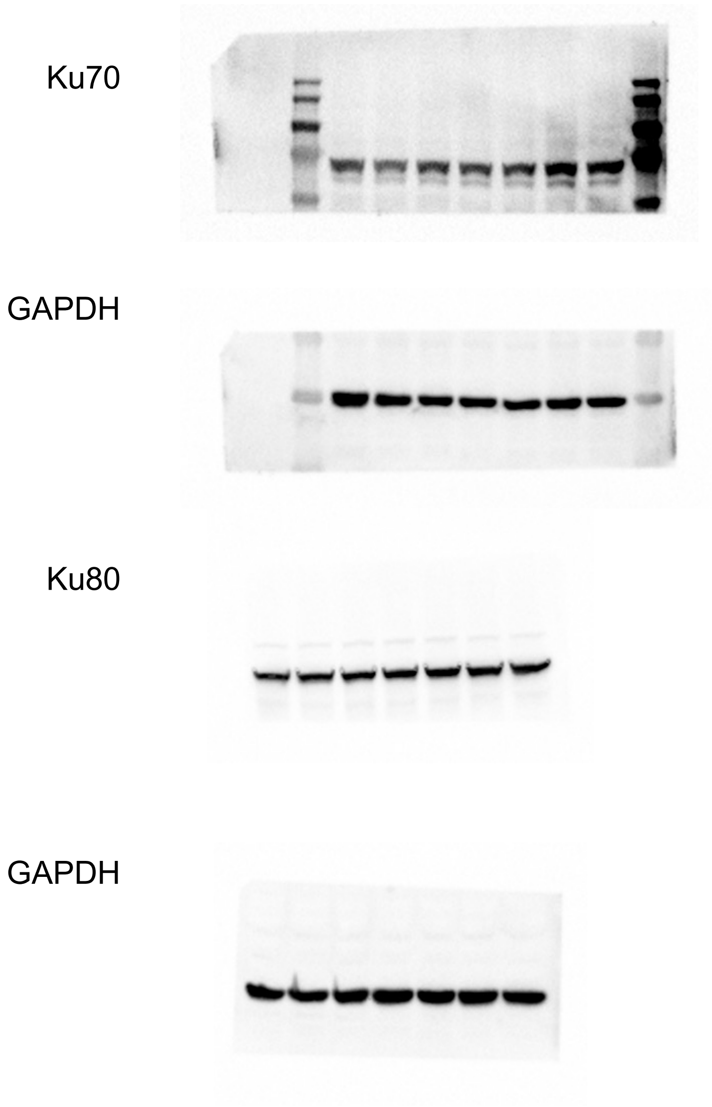

Fig. S7C 22RV1

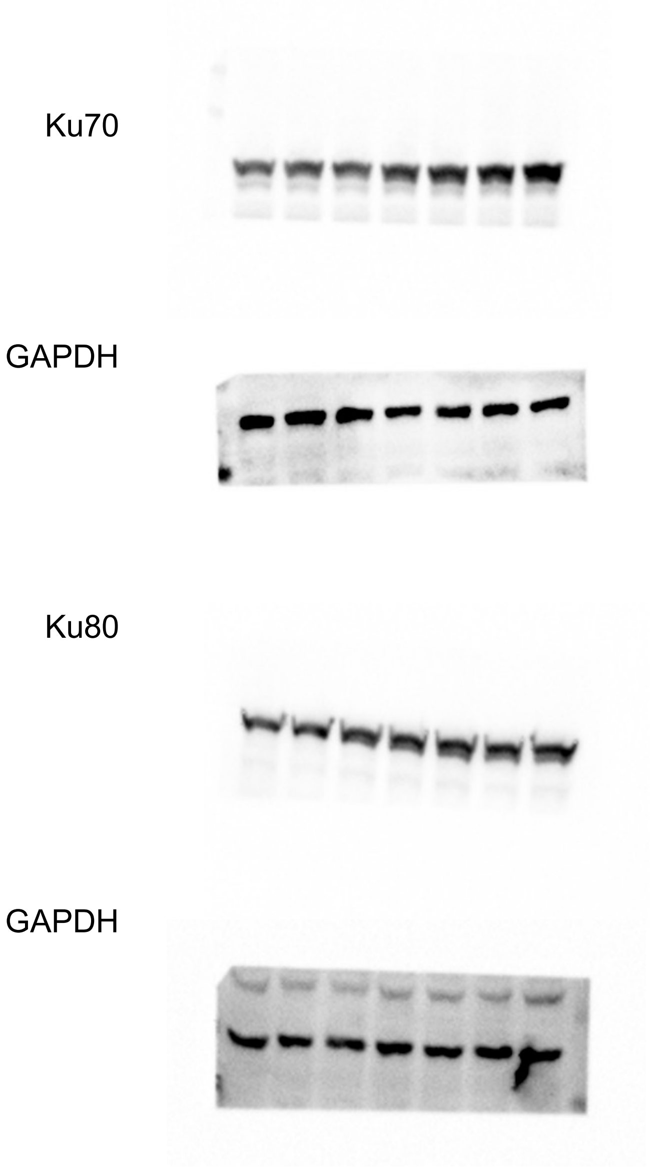

Fig. S8B C4-2

Ku80

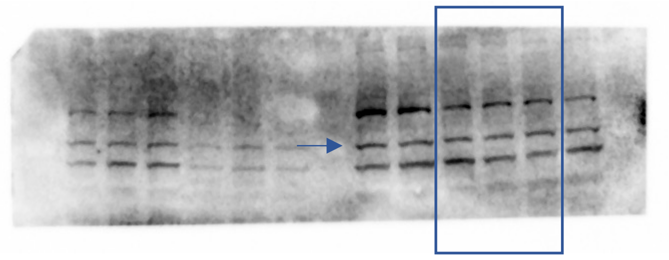

Rad51

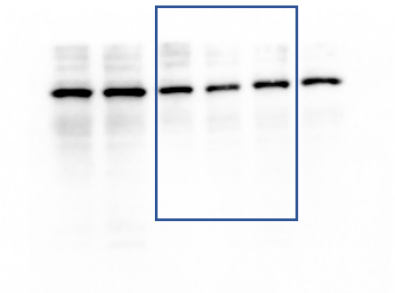

PARP

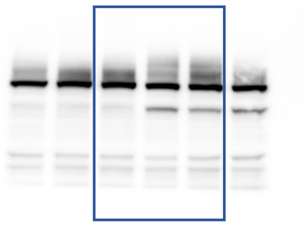

GAPDH

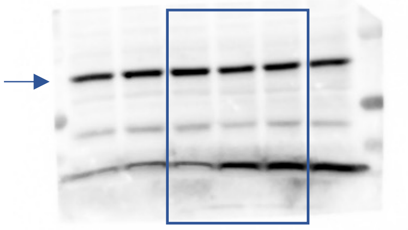

Fig. S8C C4-2

Ku80

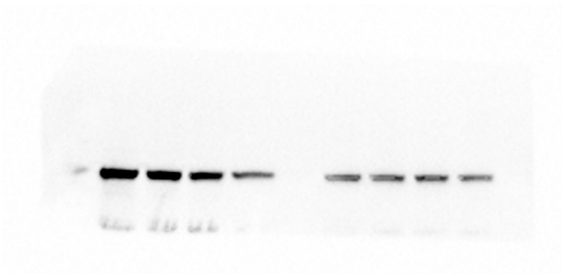

PARP

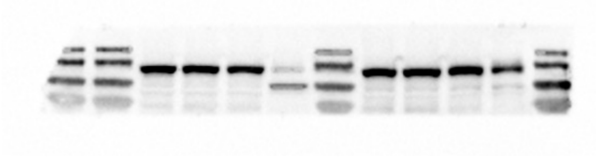

γH2A.X

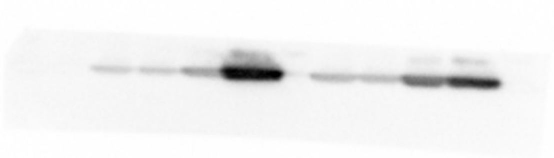

GAPDH

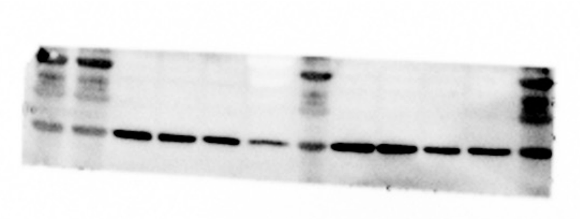

Fig. S8D DU145

Ku70

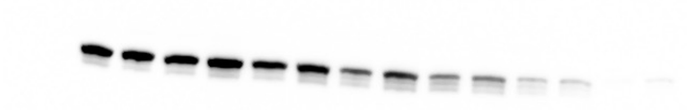

GAPDH

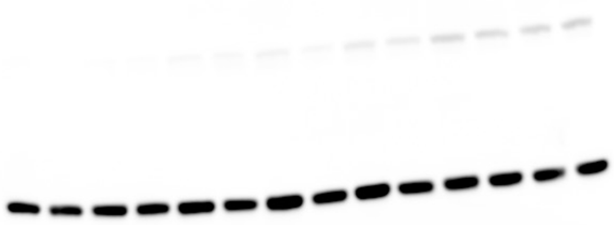

Fig. S8E DU145

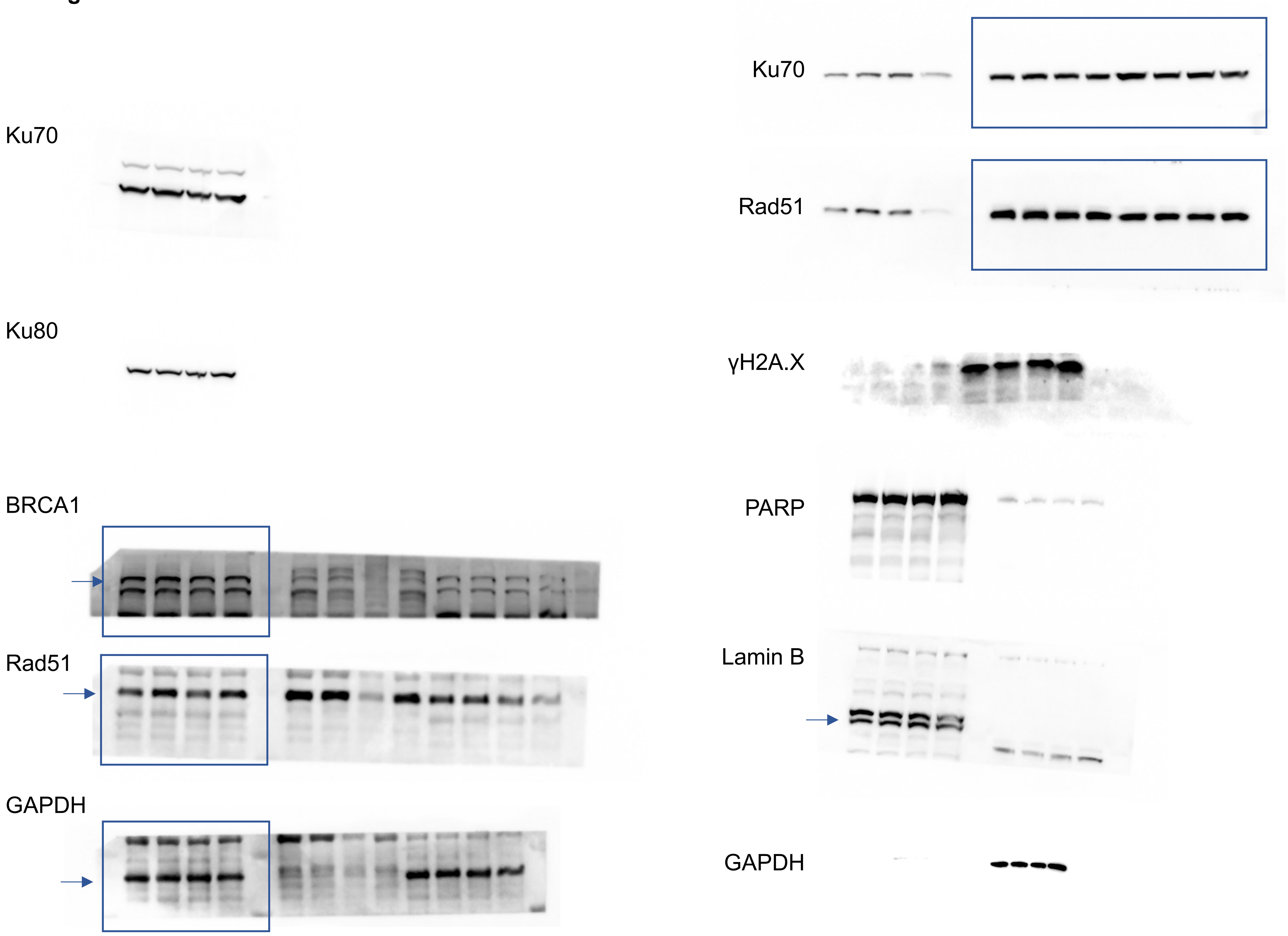

Supplement: Supplementary file 4 — Western blot original data file [file 41419_2022_5182_MOESM4_ESM.pdf]
